# Development of an explainable machine learning model for predicting depression in adults with type 2 diabetes mellitus: A cross-sectional SHAP-based analysis of NHANES 2009–2023

**DOI:** 10.1097/MD.0000000000047522

**Published:** 2026-02-06

**Authors:** Yan Tang, Lei Jia, Junjun Zhou, Jin Dou, Jingjuan Qian, Xin Yi, Kim Lam Soh

**Affiliations:** a Faculty of Nursing, Jiangsu Medical College, Yancheng, Jiangsu, China; b Department of Nursing, Faculty of Medicine and Health Sciences, University Putra Malaysia, Serdang, Selangor, Malaysia; c Department of Human Anatomy, Xiangnan University, Chenzhou, Hunan, China; d Department of Human Anatomy, Faculty of Medicine and Health Sciences, University Putra Malaysia, Serdang, Selangor, Malaysia; e Department of Endocrinology, Yancheng No. 1 People’s Hospital, Affiliated Hospital of Medical School, Nanjing University, Yancheng, China; f Department of Endocrinology, Suqian Hospital, Jiangsu Provincial People’ s Hospital, Suqian, Jiangsu, China; g Department of Internal Medicine, Faculty of Medicine and Health Sciences, Universiti Putra Malaysia, Serdang, Selangor, Malaysia.

**Keywords:** depression, machine learning, NHANES, SHapley additive exPlanations, type 2 diabetes mellitus

## Abstract

Depression (DEP) is a common yet underdiagnosed comorbidity in adults with type 2 diabetes mellitus (T2DM), worsening glycemic control and increasing complication risk. Practical, interpretable risk tools using routine patient data are limited. We conducted a cross-sectional analysis using data from adults with T2DM enrolled in the National Health and Nutrition Examination Survey between 2009 and 2023. DEP was classified based on a Patient Health Questionnaire-9 score of 10 or higher. Twenty-eight candidate predictors encompassing demographic characteristics, clinical and biochemical measurements, and lifestyle factors were initially included. Variable selection was performed using least absolute shrinkage and selection operator regression. Five machine learning algorithms – random forest, extreme gradient boosting (XGBoost), multilayer perceptron, logistic regression, and support vector machine – were trained and evaluated using 5-fold cross-validation. The best-performing model was interpreted through SHapley Additive exPlanations analysis to identify the most influential predictors. A streamlined version incorporating the top 10 predictors was further developed and implemented as a user-friendly web-based risk estimation tool. Among 2837 participants, 449 (15.8%) were identified as having comorbid DEP. The XGBoost model demonstrated the highest discriminative ability, with a validation area under the receiver operating characteristic curve of 0.888, accuracy of 0.834, F1-score of 0.715, sensitivity of 0.577, and specificity of 0.979, surpassing the performance of the other algorithms evaluated. SHapley Additive exPlanations analysis revealed gender, poverty-to-income ratio, sleep duration, smoking status, educational levels, race, age, high cholesterol, hypertension, and insulin use as the most influential predictors. A streamlined XGBoost model incorporating only these 10 variables achieved an area under the curve of 0.886, closely matching the predictive capability of the full model. The deployed web-based tool enables rapid and individualized estimation of DEP risk in patients with T2DM using routinely available clinical and demographic information. Explainable machine learning applied to nationally representative data can accurately identify adults with T2DM at heightened risk of DEP using a small set of noninvasive clinical features. The deployed tool offers a scalable, interpretable, and clinically actionable approach to support early detection and intervention, potentially improving mental health outcomes in this high-risk population.

## 1. Introduction

Type 2 diabetes mellitus (T2DM) has become a major global health challenge in the 21st century.^[1]^ According to the International Diabetes Federation Diabetes Atlas, approximately 537 million adults aged 20 to 79 years were living with diabetes in 2021, corresponding to a prevalence of 10.5%.^[2,3]^ Projections indicate that by 2045 the prevalence will increase to 12.2%, with the number of affected individuals expected to reach 783 million – around 46% higher than in 2021.^[2,3]^ Beyond its scale, T2DM is a leading cause of premature death and disability, contributing to roughly 1.66 million deaths globally in 2021, most attributable to T2DM.^[4]^ The condition is further complicated by chronic complications including cardiovascular disease,^[5]^ chronic kidney disease,^[6]^ retinopathy,^[7]^ and neuropathy,^[8]^ which substantially intensify its impact on patients and healthcare systems.

Depression (DEP) is a common mental health disorder characterized by persistent low mood, anhedonia, and frequent cognitive impairment.^[9,10]^ In 2021, an estimated 330 million people globally were living with DEP,^[11]^ which remains a major contributor to nonfatal health loss.^[12]^ The co-occurrence of DEP and T2DM is increasingly recognized as a synergistically harmful combination.^[13]^ Evidence shows that DEP in patients with T2DM accelerates disease progression,^[14]^ raises the risk of microvascular and macrovascular complications,^[15]^ impairs treatment adherence,^[16]^ and exacerbates glycemic dysregulation. These effects heighten healthcare utilization and worsen prognosis, yet many affected individuals remain undiagnosed and undertreated, stressing the need for efficient screening and risk stratification strategies.

Most existing models for classifying concurrent depressive symptoms in T2DM populations rely on conventional regression-based approaches.^[17]^ While valuable, such models often assume linear relationships and may fail to identify complex, nonlinear interactions among biological, clinical, and psychosocial predictors. As a result, their predictive performance is frequently modest. Recent advances in machine learning (ML) have shown promise in overcoming these limitations by capturing nonlinear relationships and complex interactions,^[18]^ with growing application in clinical practice to enhance identification of individuals with high likelihood of significant depressive symptoms at the time of assessment, improve diagnostic accuracy, and support individualized care.^[19]^ Moreover, interpretability techniques such as SHapley Additive exPlanations (SHAP) allow visualization of the contribution of each predictor to model output, increasing transparency and clinical utility.^[20]^

In this study, we used data from the National Health and Nutrition Examination Survey (NHANES), a nationally representative dataset comprising standardized demographic, clinical, laboratory, and lifestyle information, to develop an interpretable ML model for identifying adults with T2DM who have a high likelihood of clinically significant depressive symptoms, DEP status was determined using the Patient Health Questionnaire-9 (PHQ-9),^[21]^ which serves as a screening tool for probable DEP or clinically significant depressive symptoms. SHAP analysis was applied to determine the most influential predictors, from which a simplified, transparent model was created with the aim of enabling identification of individuals at the time of assessment with high likelihood of significant depressive symptoms and supporting timely intervention. Similar ML-based approaches have been applied in chronic kidney disease^[22]^ and cardiovascular disease^[23]^ populations to identify patients at high risk of adverse outcomes, demonstrating superior performance compared with traditional regression models and highlighting the potential utility of ML in complex clinical scenarios.

## 2. Methods and materials

### 2.1. Study population

**Figure 1.**
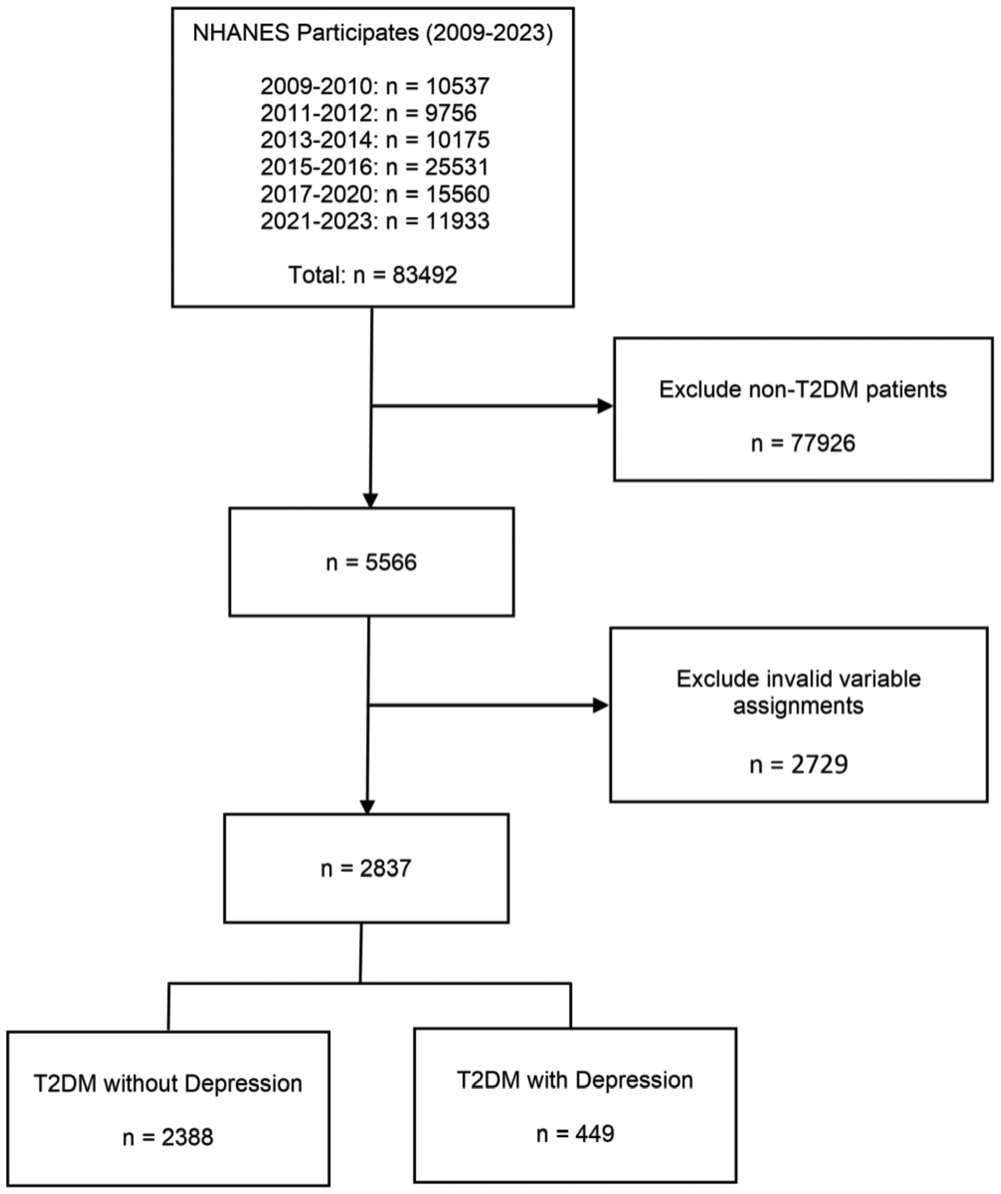
Participants screening process. NHANES = National Health and Nutrition Examination Survey, T2DM = type 2 diabetes mellitus.

This cross-sectional study analyzed data from the NHANES collected between 2009 and 2023, comprising a total of 83,492 participants. We first excluded individuals without T2DM (n = 77,926) and those with invalid or missing values for key study variables (n = 2729). After applying these criteria, a total of 2837 participants with complete data were included in the final analysis. Among them, 449 participants had comorbid DEP (T2DM with DEP group), and 2388 participants did not (T2DM without DEP group). The detailed screening process is shown in Figure 1.

### 2.2. Definition of T2DM and DEP

T2DM was defined based on participants’ self-reported medical history, specifically those who answered “Yes” to the question “Have a doctor or health professional ever told you that you have diabetes?” and were aged ≥ 20 years.

Depressive symptoms were evaluated using the PHQ-9, a validated 9-item self-report instrument designed to screen for depressive symptoms over the preceding 2 weeks.^[24]^ Each item addresses one of the core criteria for major depressive disorder according to the Diagnostic and Statistical Manual of Mental Disorders, Fourth Edition,^[25]^ including:

1. Little interest or pleasure in doing things?
2. Feeling down, depressed, or hopeless?
3. Trouble sleeping or sleeping too much?
4. Feeling tired or having little energy?
5. Poor appetite or overeating?
6. Feeling bad about oneself?
7. Trouble concentrating on things?
8. Moving or speaking slowly, or being fidgety/restless?
9. Thoughts of self-harm or that one would be better off dead?

Responses for each item were scored from 0 to 3, corresponding to “not at all,” “several days,” “more than half the days,” and “nearly every day,” respectively. Participants endorsing any of the 9 symptoms were further asked about the degree to which these problems impaired their daily functioning.

For each participant, a total PHQ-9 score was calculated by summing the item scores. Participants with a total score ≥ 10 were considered to have clinically significant depressive symptoms. Based on this criterion, the T2DM population was divided into 2 groups for analysis: T2DM with DEP and T2DM without DEP, reflecting concurrent depressive symptoms at the time of assessment.

### 2.3. Clinical variable selection and processing

In this study, we initially considered 32 clinical variables from the NHANES database to capture demographic characteristics, anthropometric measurements, hematologic and biochemical parameters, as well as disease history and lifestyle factors. For categorical variables, records with invalid or missing values were directly excluded. For continuous variables, those with a missing rate ≥ 20% were removed. After data cleaning and processing, a total of 28 variables were ultimately included in the analysis. These comprised demographic characteristics: gender, age, race, education, and family income-to-poverty ratio (PIR); anthropometric measurements: height, weight, and body mass index (BMI); hematologic and biochemical parameters: high-density lipoprotein cholesterol, white blood cell count, lymphocyte percentage, monocyte percentage, neutrophil percentage, eosinophil percentage, basophil percentage, red blood cell count, hemoglobin, hematocrit, red cell distribution width, platelet count, mean platelet volume; disease history: hypertension and high cholesterol; diabetes-related medication use: taking diabetic pills and taking insulin; and lifestyle factors: sleeping duration per day (Sleep_D) and smoking status. Variables with a missing rate < 20% were subjected to multiple imputation by chained equations to minimize bias and retain maximal sample information. Imputation was performed on these 28 variables a total of 5 times before splitting the data into training and validation sets. The first imputed dataset was then used for subsequent model training and evaluation. All variables and their measurement protocols can be accessed on the official NHANES website (https://www.cdc.gov/nchs/).

### 2.4. ML model development and validation

For model development, 28 clinical variables were initially considered. To reduce potential multicollinearity and prevent overfitting, we applied the least absolute shrinkage and selection operator regression, which performs both variable selection and regularization, retaining only the most informative predictors. Class imbalance in the outcome variable was addressed using the synthetic minority over-sampling technique, ensuring that the minority class (T2DM with DEP group) was adequately represented in the training set.

In this study, 5 ML algorithms, namely random forest (RF), extreme gradient boosting (XGBoost), support vector machine (SVM), multilayer perceptron (MLP), and logistic regression (LOG), were applied to build predictive models assessing the risk of DEP in patients with T2DM. The complete dataset was randomly divided into a training set comprising 70% of the cases and a validation set containing the remaining 30%. During the model development stage, 5-fold cross-validation was conducted within the training set. This procedure enabled refinement of hyperparameters, enhanced the ability of the models to generalize beyond the training data, and reduced the likelihood of overfitting. Model performance was examined using the independent validation set, with evaluation based on multiple indicators including the area under the curve (AUC) the receiver operating characteristic curve, accuracy, sensitivity, specificity, and the F1-score, thereby providing a comprehensive assessment of predictive capacity. The algorithm that demonstrated superior and consistent results across all evaluation metrics was identified as the optimal model and reserved for subsequent detailed analysis.

### 2.5. Model simplification, interpretation, and deployment

To improve the interpretability of the selected ML model and to support its potential use in clinical settings, SHAP analysis was conducted. The SHAP importance values for each variable were calculated to quantify their relative contributions to the model’s predictions. Variables were ordered according to these importance scores, and the 10 predictors with the highest contributions were identified as the most influential factors. Following this step, SHAP dependence plots were examined to characterize both the direction and the magnitude of each selected variable’s association with the predicted risk of DEP in patients with T2DM. This analysis provided a visual and quantitative understanding of how changes in individual features impact the likelihood of comorbid DEP.

Using the 10 most influential predictors, a streamlined XGBoost model was developed. Performance evaluation for this simplified model was carried out using the same metrics applied to the original analysis: area under the AUC, accuracy, sensitivity, specificity, and the F1-score. The results indicated that, despite the reduction in the number of input features, the simplified model maintained a high level of predictive accuracy. This finding suggests that the most important risk factors identified by SHAP accounted for the majority of the predictive information present in the full set of variables.

Finally, this simplified model was deployed as a web-based interactive application, enabling clinicians to input patient-specific information and obtain individualized risk predictions for comorbid DEP in T2DM patients. This tool provides a user-friendly and clinically actionable platform to support decision-making in routine practice.

### 2.6. Statistical analysis

All analysis were performed using R software (version 4.5., R Foundation for Statistical Computing, Vienna, Austria). Continuous variables were first assessed for normality using the Kolmogorov–Smirnov test and skewness. Variables showing no significant deviation from normality (*P* > .05) or with skewness ≤ 1 were summarized as mean ± standard deviation, whereas those with significant deviation from normality and skewness > 1 were summarized as median with interquartile range. A *P* value < .05 was considered statistically significant.

## 3. Results

### 3.1. Baseline characteristics of T2DM participants with and without DEP

A total of 2837 participants with T2DM were included, of whom 449 had comorbid DEP. Participants with DEP were younger and had lower PIR, higher BMI and weight, and slightly shorter height compared with those without DEP (all *P* < .05). Most laboratory measurements, including HDL, lymphocyte percentage, neutrophil percentage, monocyte percentage, eosinophil percentage, basophil percentage, and Glu, were similar between groups. Participants with DEP showed modestly higher white blood cell count, hemoglobin, hematocrit, red cell distribution width, platelet count, and mean platelet volume compared with nondepressed participants (all *P* < .05), whereas daily sleep duration was slightly shorter (*P* = .022). In categorical variables, DEP was more prevalent among females, while males were less represented in the DEP group (*P* < .001). Race/ethnicity distributions differed slightly between groups (*P* = .017), with higher proportions of Mexican American and Other Hispanic participants and slightly lower proportions of Non-Hispanic Black and Other Race participants in the DEP group. Participants with DEP were more likely to have lower educational attainment, including < 9th grade or 9 to 11th grade, and were less likely to be college graduates (*P* < .001). Regarding comorbidities, hypertension and high cholesterol were more common in participants with DEP (both *P* = .003), whereas insulin use was slightly higher (*P* = .026) and oral diabetic medication use did not differ significantly (*P* = .120). Smoking prevalence was higher in the DEP group (*P* = .003). As shown in Table 1.

**Table 1:** Baseline characteristics of T2DM participants with and without DEP.

Variable	Overall (n = 2837)	T2DM without DEP (n = 2388)	T2DM with DEP(n = 449)	*P*
Continuous variables, mean ± SD/Median (IQR)				
Age (yr)	61.41 ± 12.52	61.88 ± 12.51	58.87 ± 12.29	**<.001**
PIR	2.28 ± 1.54	2.39 ± 1.57	1.65 ± 1.22	**<.001**
Height (cm)	166.35 ± 10.38	166.75 ± 10.33	164.22 ± 10.39	**<.001**
Weight (kg)	88.00 (74.20–104.70)	87.35 (73.90–104.10)	90.90 (76.30–107.00)	**.014**
BMI (kg/m^2^)	31.50 (27.47–36.90)	31.18 (27.29–36.39)	33.40 (29.00–39.00)	**<.001**
HDL (mmol/L)	1.19 (1.01–1.42)	1.19 (1.01–1.42)	1.19 (0.98–1.40)	.638
WBC (×10^3^/µL)	7.59 ± 2.25	7.55 ± 2.22	7.82 ± 2.35	**.027**
Lymph (%)	29.09 ± 8.88	29.13 ± 8.88	28.89 ± 8.91	.604
Mono (%)	7.60 (6.40–9.20)	7.60 (6.40–9.20)	7.50 (6.20–9.10)	.146
Neut (%)	59.40 ± 9.56	59.33 ± 9.54	59.79 ± 9.71	.359
Eos (%)	2.50 (1.60–3.70)	2.40 (1.60–3.70)	2.50 (1.60–3.70)	.969
Baso (%)	0.70 (0.50–1.00)	0.70 (0.50–1.00)	0.80 (0.50–1.00)	.657
RBC (×10^6^/µL)	4.61 ± 0.55	4.62 ± 0.55	4.57 ± 0.55	.068
HGB (g/dL)	13.63 ± 1.62	13.67 ± 1.62	13.45 ± 1.64	**.011**
HCT (%)	40.60 ± 4.55	40.68 ± 4.53	40.13 ± 4.68	**.021**
RDW (%)	13.70 (13.10–14.60)	13.70 (13.10–14.50)	13.80 (13.10–14.80)	**.029**
PLT (×10^3^/µL)	237.80 ± 71.48	236.21 ± 69.41	246.23 ± 81.15	**.014**
MPV (fL)	8.39 ± 0.98	8.36 ± 0.96	8.53 ± 1.07	**.002**
Glu (mmol/L)	6.90 (6.20–8.20)	6.90 (6.20–8.20)	6.90 (6.20–8.60)	.493
Sleep_D (h)	7.15 ± 1.91	7.19 ± 1.81	6.92 ± 2.39	**.022**
Categorical variables, n (%)				
Gender				
Male	1454 (51.3%)	1289 (54.0%)	165 (36.7%)	**<.001**
Female	1383 (48.7%)	1099 (46.0%)	284 (63.3%)	
Race				
Mexican American	396 (14.0%)	330 (13.8%)	66 (14.7%)	**.017**
Other Hispanic	325 (11.5%)	256 (10.7%)	69 (15.4%)	
Non-Hispanic White	1017 (35.8%)	853 (35.7%)	164 (36.5%)	
Non-Hispanic Black	770 (27.1%)	660 (27.6%)	110 (24.5%)	
Other Race	329 (11.6%)	289 (12.1%)	40 (8.9%)	
Education				
<9th grade	387 (13.6%)	309 (12.9%)	78 (17.4%)	**<.001**
9–11th grade	442 (15.6%)	341 (14.3%)	101 (22.5%)	
High school/GED	646 (22.8%)	552 (23.1%)	94 (20.9%)	
Some college/AA	885 (31.2%)	742 (31.1%)	143 (31.8%)	
College graduate	477 (16.8%)	444 (18.6%)	33 (7.3%)	

Variable	Overall (n = 2106)	T2DM without DEP (n = 1604)	T2DM with DEP(n = 502)	*P*
Hypertension				
Yes	2038 (71.8%)	1689 (70.7%)	349 (77.7%)	**.003**
No	799 (28.2%)	699 (29.3%)	100 (22.3%)	
High cholesterol				
Yes	1870 (65.9%)	1546 (64.7%)	324 (72.2%)	**.003**
No	967 (34.1%)	842 (35.3%)	125 (27.8%)	
Taking insulin				
Yes	827 (29.2%)	676 (28.3%)	151 (33.6%)	**.026**
No	2010 (70.8%)	1712 (71.7%)	298 (66.4%)	
Taking diabetic pills				
Yes	2023 (71.3%)	1717 (71.9%)	306 (68.2%)	.120
No	814 (28.7%)	671 (28.1%)	143 (31.8%)	
Smoking				
Yes	1400 (49.3%)	1149 (48.1%)	251 (55.9%)	**.003**
No	1437 (50.7%)	1239 (51.9%)	198 (44.1%)	**.003**

Baso = basophil, BMI = body mass index, DEP = depression, Eos = eosinophil, Glu = glucose, HCT = hematocrit, HDL = high-density lipoprotein cholesterol, HGB = hemoglobin, IQR = interquartile range, Lymph = lymphocyte, Mono = monocyte, MPV = mean platelet volume, Neut = neutrophil, PIR = family income-to-poverty ratio, PLT = platelet count, RBC = red blood cell count, RDW = red cell distribution width, SleepD = sleeping duration per day, T2DM = type 2 diabetes mellitus, WBC = white blood cell count. Bold values indicate statistical significance (*P* < .05).

### 3.2. Comparison of ML models and selection of the optimal predictor

We systematically compared the predictive performance of 5 ML algorithms – RF, XGBoost, MLP, LOG, and SVM – for DEP in patients with T2DM. In the training set, XGBoost exhibited the highest discriminative ability, achieving an AUC of 0.912 (95% CI 0.900–0.924), with a balanced overall performance: accuracy 0.852, F1-score 0.746, sensitivity 0.603, and specificity 0.992. RF and MLP also demonstrated substantial predictive capability, though slightly lower than XGBoost (RF AUC 0.895, MLP AUC 0.856), while LOG and SVM showed moderate discrimination (AUCs 0.728 and 0.737, respectively), indicating limited ability to identify DEP cases in this cohort. In the validation set, XGBoost maintained robust performance, with an AUC of 0.888 (95% CI 0.867–0.909), accuracy 0.834, F1-score 0.715, sensitivity 0.577, and specificity 0.979, demonstrating stable generalization. RF showed slightly lower performance compared with XGBoost, whereas LOG and SVM exhibited comparatively modest discriminative ability.

**Figure 2.**
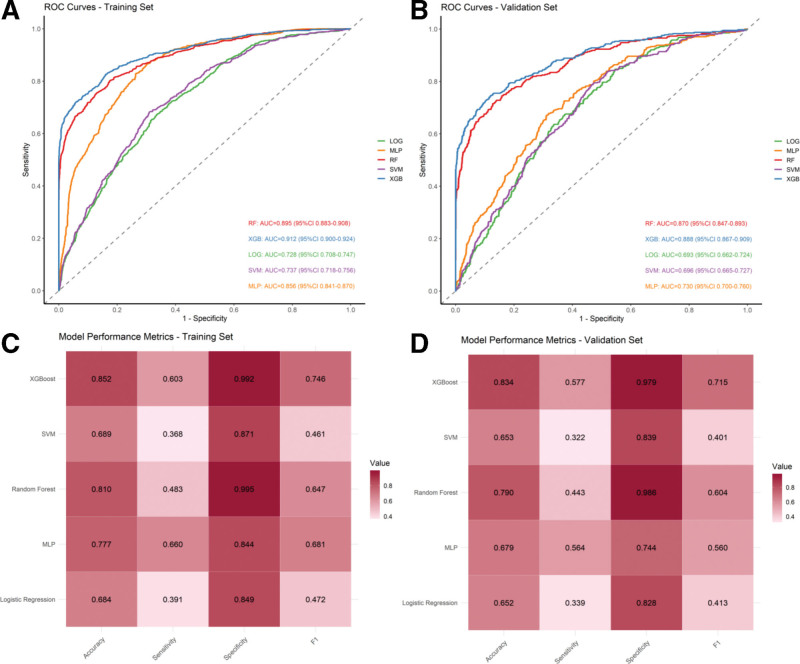
ROC curves and confusion matrices for 5 ML models (A: ROC curve in training set; B: ROC curve in validation set; C: Confusion metric in training set; and D: Confusion metric in validation set). ML = machine learning, ROC = receiver operating characteristic curve.

Collectively, these results highlight the superiority of ensemble tree-based methods for DEP risk stratification in T2DM, with XGBoost demonstrating the optimal combination of discrimination, calibration, and robustness. Its superior performance can be attributed to its ability to capture complex nonlinear relationships, handle feature interactions effectively, and maintain stability across training and validation sets. Therefore, XGBoost was selected as the optimal predictive model for subsequent analysis. As shown in Figure 2.

### 3.3. SHAP analysis of the optimal XGBoost model

**Figure 3.**
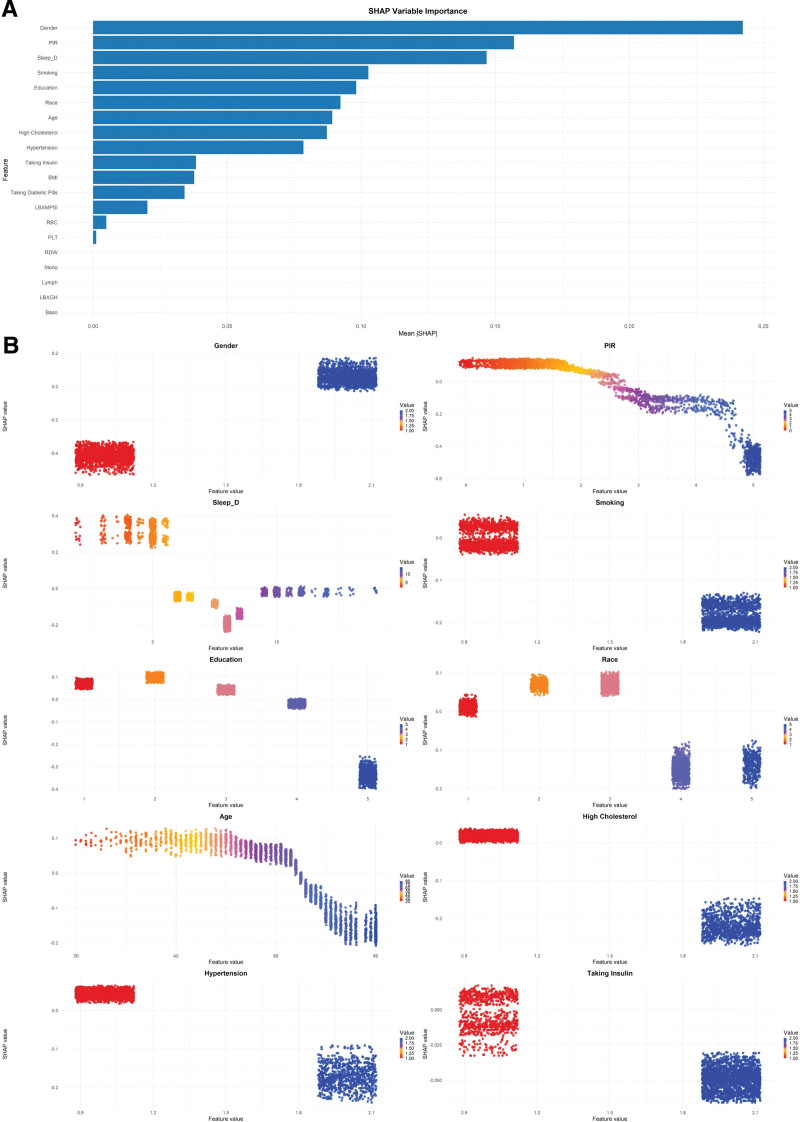
SHAP analysis of the optimal XGBoost model (A: SHAP importance plot for all variables; B: SHAP dependence plots for top 10 variables; Gender: 1 = Male, 2 = Female; Hypertension, High Cholesterol, Smoking, and Taking Insulin: 1 = Yes, 2 = No; Race: 1 = Mexican American; 2 = Other Hispanic; 3 = Non-Hispanic 4 = White; 5 = Non-Hispanic Black; and 6 = Other Race; Education: 1 = <9th grade; 2 = 9 to 11th grade; 3 = High school/GED; 4 = Some college/AA; and 5 = College graduate). SHAP = SHapley additive exPlanations, XGBoost = extreme gradient boosting.

To further elucidate the contribution of individual features to the prediction of comorbid DEP in patients with T2DM, we applied SHAP to the optimal XGBoost model. Initially, variable importance was ranked according to the mean absolute SHAP values, and the top 10 predictors were selected for detailed interpretation using SHAP dependence plots, as shown in Figure 3A. The top-ranked features, in descending order of importance, were: gender (0.242), PIR (0.157), Sleep_D (0.147), smoking (0.103), education (0.098), race (0.092), age (0.089), high cholesterol (0.087), hypertension (0.078), and taking insulin (0.038).

For PIR, higher values were associated with progressively lower SHAP values, indicating a shift in the model prediction towards the nondepressed state and suggesting a reduced risk of DEP with increasing PIR. Regarding age, SHAP values remained relatively high and stable before approximately 62 years, corresponding to an elevated predicted risk of DEP; beyond this threshold, a sharp decline in SHAP values was observed, indicating a pronounced reduction in DEP risk in older individuals. Sleep_D demonstrated a nonlinear relationship with DEP risk: values < 6 hours were associated with the highest SHAP values and, consequently, the greatest predicted risk. Between 6 and 8 hours, SHAP values markedly declined, indicating a protective effect of adequate sleep, whereas durations ≥ 9 to 10 hours were again linked with elevated SHAP values, reflecting increased risk. Moreover, our streamlined model, which includes only 10 variables, retained substantial discriminative performance, highlighting the efficiency of SHAP-guided feature selection and demonstrating the model’s readiness for real-time, low-burden clinical implementation.

Regarding education, participants with <9th grade and 9 to 11th grade education exhibited higher SHAP values, indicating the highest predicted risk of DEP among T2DM patients. In contrast, as educational attainment increased, DEP risk progressively declined, reaching the lowest predicted risk among those with a college graduate. Concerning Race, Non-Hispanic White participants had the highest SHAP values, whereas Mexican American and Other Hispanic individuals showed slightly lower but still relatively high SHAP values, suggesting elevated DEP risk in these 3 groups. By contrast, Non-Hispanic Black and Other Race participants had low SHAP values, indicating a lower predicted risk of DEP. For treatment-related variables, taking insulin demonstrated a complex relationship with predicted DEP risk in the SHAP analysis. Patients who answered “Yes” exhibited predominantly moderate-to-high SHAP values, suggesting that insulin use was associated with an elevated predicted risk of comorbid DEP. In contrast, those who responded “No” generally showed low-to-moderate SHAP values, indicating a lower predicted risk among non-insulin users. Notably, there was partial overlap in SHAP values between the 2 groups, highlighting that the effect of insulin use on DEP risk may be context-dependent and potentially bidirectional under different clinical conditions. In addition, “Yes” responses for smoking, hypertension, and high cholesterol were consistently linked with higher SHAP values, indicating an increased likelihood of comorbid DEP in these patients. Regarding gender, female participants exhibited notably higher SHAP values compared to males, suggesting a substantially greater risk of DEP among women with T2DM, as shown in Figure 3B.

### 3.4. XGBoost model simplification and deployment

To enhance applicability in clinical practice, we retrained a streamlined XGBoost model that incorporated only the top 10 features identified by SHAP analysis. This reduction in input variables offers several advantages: it lowers the burden of data collection, shortens computation time, improves interpretability, and facilitates integration into real-time clinical workflows.

**Figure 4.**
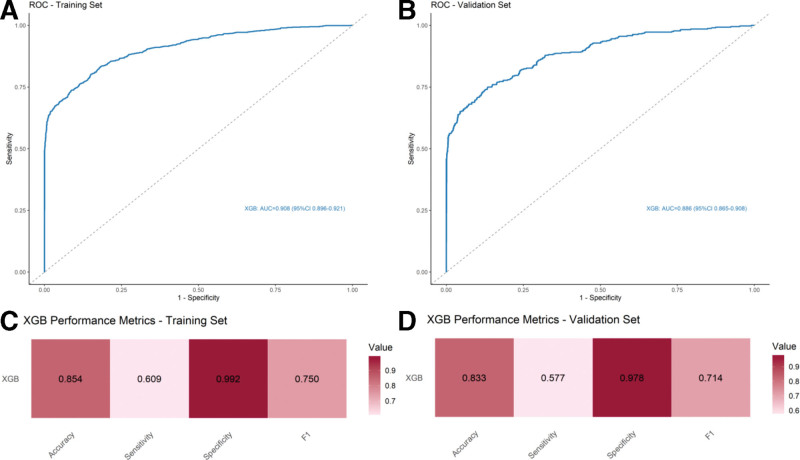
ROC curves and confusion matrices for simplified XGBoost models (A) ROC curve in training set; (B) ROC curve in validation set; (C) confusion metric in training set; and (D) confusion metric in validation set. ROC = receiver operating characteristic curve, XGBoost = extreme gradient boosting.

On the training set, the simplified model achieved an AUC of 0.908 (95% CI: 0.896–0.921), accuracy of 0.854, sensitivity of 0.609, specificity of 0.992, and an F1-score of 0.750. Performance on the validation set was comparably strong, with an AUC of 0.886 (95% CI: 0.865–0.908), accuracy of 0.833, sensitivity of 0.577, specificity of 0.978, and an F1-score of 0.714. These metrics were closely aligned with those of the full-variable XGBoost model (validation AUC = 0.888, accuracy = 0.834, sensitivity = 0.577, specificity = 0.979, and F1-score = 0.715), indicating that predictive power was largely preserved despite the reduction in model complexity. As shown in Figure 4.

**Figure 5.**
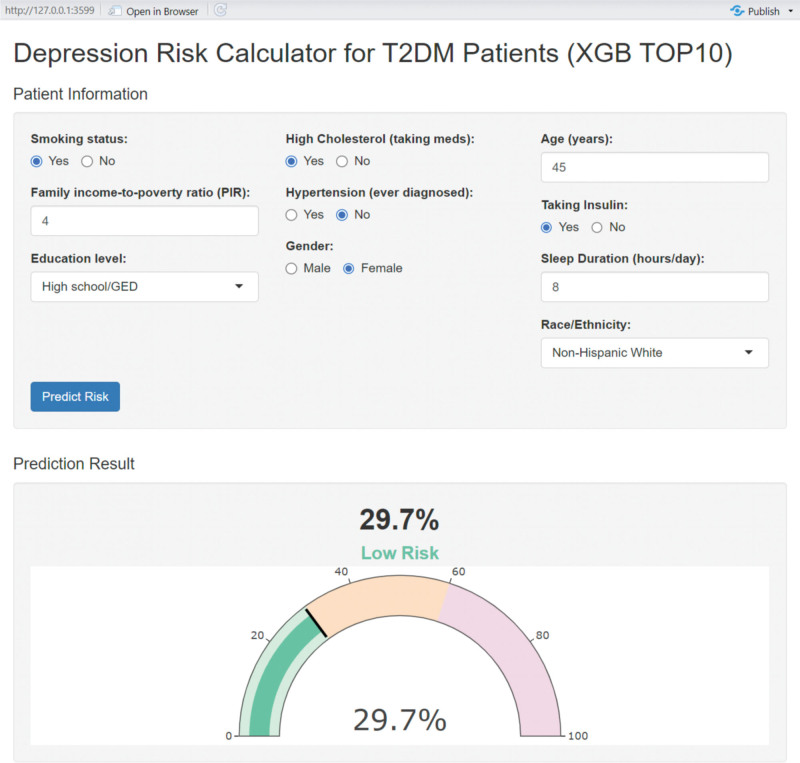
Web-based interactive application for risk prediction of comorbid DEP in T2DM patients. DEP = depression, T2DM = type 2 diabetes mellitus.

Given its robust performance, the simplified model was subsequently deployed as a web-based application. This tool enables clinicians to input a limited set of clinical and demographic variables and obtain an individualized estimate of high DEP risk among patients with T2DM, supporting early identification and timely intervention. As shown in Figure 5. The online application is publicly accessible for real-time use at https://eyu666.shinyapps.io/dep_risk_app.

## 4. Discussion

This study developed and validated an explainable ML approach to identify adults with T2DM who have a high likelihood of clinically significant depressive symptoms, achieving high discrimination while retaining interpretability through SHAP analysis. Among the 5 ML algorithms evaluated, XGBoost provided the best overall performance, and a streamlined variant using only 10 top-ranked predictors achieved nearly identical results, demonstrating the feasibility of a clinically efficient model.

When compared with prior work^[17]^ based on multiple LOG, both our full-variable and streamlined XGBoost models demonstrated superior predictive performance for identifying individuals with high likelihood of clinically significant depressive symptoms in adults with T2DM across all major evaluation metrics. In the referenced regression study, the training set achieved a sensitivity of 62.4%, specificity of 80.0%, accuracy of 77.6%, and an AUC of 0.780, while the testing set yielded 65.6% sensitivity, 75.4% specificity, 74.2% accuracy, and an AUC of 0.752. In contrast, our full-variable XGBoost model reached, in the training set, an AUC of 0.912, accuracy of 85.2%, sensitivity of 60.3%, specificity of 99.2%, and F1-score of 0.746; and in the validation set, an AUC of 0.888, accuracy of 83.4%, sensitivity of 57.7%, specificity of 97.9%, and F1-score of 0.715. The streamlined 10-predictor XGBoost performed almost identically, achieving a training set AUC of 0.908, accuracy of 85.4%, sensitivity of 60.9%, specificity of 99.2%, and F1-score of 0.750; and a validation set AUC of 0.886, accuracy of 83.3%, sensitivity of 57.7%, specificity of 97.8%, and F1-score of 0.714. Notably, while sensitivity values across our models were on par with the LOG benchmark, specificity and AUC were consistently and substantially higher, indicating more accurate separation of depressed and nondepressed cases. Moreover, our streamlined model, which includes only 10 variables, retained substantial discriminative performance, highlighting the efficiency of SHAP-guided feature selection and demonstrating the model’ s readiness for real-time, low-burden clinical implementation. However, the relatively moderate sensitivity indicates that a proportion of individuals with clinically significant depressive symptoms may be missed. This trade-off between high specificity and moderate sensitivity should be considered when applying the model for screening or case-finding purposes, and complementary strategies may be needed to minimize false negatives in clinical practice.

SHAP analysis identified female sex, poverty-to-income ratio, sleep duration, smoking status, educational attainment, race, age, hypercholesterolemia, hypertension, and insulin use as the most influential predictors of DEP in T2DM. Several of these overlapped with variables included in prior multiple LOG analysis,^[18]^ namely age, gender, PIR, BMI, educational attainment, smoking status, LDL-C, sleep duration, and sleep disorder, indicating a degree of consistency across methods, yet with notable differences in both variable inclusion and relationship patterns.

In patients with T2DM, the risk of DEP appears to be closely associated with demographic factors, particularly sex and age. Female patients showed a higher likelihood of depressive symptoms. Regarding age, the highest risk was noted in individuals aged 20 to 62 years, consistent with previous evidence that earlier or middle-age onset of T2DM is more strongly linked to the incidence and severity of depressive symptoms.^[26]^ Patients in this age range often face substantial occupational and family responsibilities, and the long-term burden of disease management heightens psychological stress.^[27]^ diagnosis at this stage also implies longer disease duration and greater cumulative risk of complications, triggering anxiety and DEP.^[28]^ Furthermore, persistent reductions in quality of life and self-efficacy serve as important psychological mechanisms underlying DEP in these individuals.^[29,30]^ By contrast, in older age, the alleviation of work-related stress and expansion of social networks may partially offset depressive risk.

Socio-cultural factors also emerged as significant determinants of DEP risk in individuals with T2DM. Socioeconomic disadvantage, in particular, exerts its influence through both neuroendocrine and psychosocial pathways. Chronic financial strain and social adversity have been associated with dysregulation of the hypothalamic–pituitary–adrenal axis and abnormal cortisol dynamics, which may impair hippocampal neuroplasticity and disrupt glucocorticoid feedback sensitivity.^[31,32]^ In T2DM, such hypothalamic–pituitary–adrenal alterations can aggravate insulin resistance and inflammatory activity, further linking metabolic disturbances to mood disorders.^[33]^ Psychosocial mechanisms, including restricted access to stable housing, nutritious food, healthcare, and supportive social networks, impose sustained stress and increase psychological burden, thereby amplifying vulnerability to DEP.^[34,35]^ Consistent with previous reports,^[36]^ our findings indicate that patients with lower educational attainment are at higher risk, possibly due to reduced health literacy and diminished capacity to understand and implement effective self-management strategies,^[37]^ leading to suboptimal glycemic control, more frequent complications, and heightened psychological distress.^[38,39]^ Ethnic differences were also observed: individuals identified as “Other Hispanic” and “Non-Hispanic White” had the highest DEP prevalence, with Mexican Americans also showing elevated rates. These disparities may reflect the combined impact of socioeconomic position, inequitable access to healthcare, and variations in cultural norms and social support resources.^[40]^

To ensure responsible clinical use of the model, careful consideration of algorithmic fairness is essential. Socioeconomic and racial/ethnic variables strongly influence predictions, which may inadvertently encode structural inequities present in the population. Without appropriate safeguards, such patterns could disproportionately label certain groups as “high risk,” potentially reinforcing existing disparities. Incorporating fairness audits, evaluating subgroup performance, and ensuring contextual interpretation of predictions are therefore critical steps before clinical deployment. These measures help ensure that the model supports equitable care rather than amplifying inequities.

Lifestyle factors were also significantly associated with DEP risk in patients with T2DM. Sleep duration demonstrated a U-shaped relationship with depressive symptoms, whereby both insufficient and prolonged sleep were linked to greater risk. Short sleep may disrupt circadian rhythms, heighten sympathetic nervous system activity, elevate nocturnal cortisol secretion, and reduce slow-wave sleep, ultimately impairing glucose (Glu) homeostasis and emotional regulation.^[41,42]^ Conversely, excessive sleep has also been associated with increased DEP risk, although the magnitude of risk appears lower than that observed with sleep deficiency.^[43,44]^ In contrast, maintaining approximately 6 to 8 hours of sleep per night seems to favor circadian rhythm alignment, a balanced architecture of rapid eye movement and slow-wave sleep, and stable neuroendocrine function, thereby enhancing resilience to depressive symptoms. In our study, smoking was likewise associated with higher DEP risk among individuals with T2DM. Mechanistically, nicotine may acutely stimulate dopamine, serotonin, and norepinephrine release, transiently elevating mood, but chronic exposure results in receptor down-regulation and neurotransmitter depletion, thereby increasing vulnerability to mood disorders.^[45,46]^ Moreover, smoking in T2DM accelerates endothelial dysfunction and oxidative stress, further contributing to depressive risk.^[47,48]^ Elevated inflammatory markers have also been observed in patients with comorbid T2DM and DEP,^[49]^ and given that smoking is often accompanied by unhealthy lifestyle behaviors and poor glycemic control, these factors may synergistically amplify psychological burden and depressive susceptibility.^[50]^ We also found that patients with T2DM receiving insulin therapy exhibited an overall higher risk of DEP, consistent with findings from previous studies.^[51]^ This association may be partly explained by the fact that insulin users typically have longer disease duration, more advanced disease severity, and heavier psychological burden.^[52]^ In addition, the demands of intensive blood Glu monitoring and injection management may further increase treatment-related stress. Interestingly, overlapping SHAP values between insulin users and nonusers suggest a bidirectional effect: in certain populations, insulin therapy may improve glycemic control, stabilize disease progression, and thereby mitigate depressive symptoms.^[15]^

In patients with T2DM, the presence of hypercholesterolemia or hypertension is associated with a markedly elevated risk of DEP. These vascular–metabolic risk factors may contribute to depressive vulnerability by distinct but converging mechanisms: hypercholesterolemia promotes atherosclerotic processes, whereas hypertension accelerates microvascular injury. Both pathways impair cerebral blood flow regulation and neurovascular coupling, thereby reducing metabolic support to the prefrontal cortex and limbic circuits that are critically involved in mood regulation.^[53,54]^ Moreover, the cumulative burden of multiple chronic comorbidities represents an additional psychosocial risk factor for DEP. As the number of coexisting conditions increases, patients often experience heightened daily stress and treatment demands, which further amplify the likelihood of depressive symptoms.^[55]^

By incorporating biological, behavioral, socioeconomic, and treatment-related factors into an interpretable ML framework, this study demonstrates that explainable ML can achieve predictive accuracy comparable to – and in certain aspects exceeding – that of conventional epidemiological models. Using a concise set of routinely collected, noninvasive variables, our approach enabled high-performance, identifying individuals with high likelihood of clinically significant depressive symptoms in adults with T2DM. The model integrates sex hormone-related neurobiological mechanisms, lifestyle patterns, social determinants, and treatment burden into a transparent structure, providing clinically meaningful and actionable insights. Compared with routine PHQ-9 screening, this approach provides complementary benefits by enabling personalized risk assessment, highlighting patient-specific risk factors, and guiding targeted interventions, thereby supporting informed clinical decision-making and individualized care strategies.

Several limitations should be noted. First, the cross-sectional design limits causal inference; longitudinal research is required to clarify temporal dynamics and potential bidirectional associations between diabetes, its determinants, and DEP. Second, DEP status was based on the PHQ-9 self-report instrument rather than structured clinical interviews, introducing possible misclassification despite its broad validation in epidemiological studies. Third, while internal validation indicates robustness, external validation in independent, prospective, multicenter cohorts is necessary to verify generalizability across diverse healthcare systems, cultural contexts, and demographic groups. Additionally, psychosocial factors such as life events, coping styles, and social support were not available in the NHANES dataset but may further refine model performance. Future work should explore the integration of multi-source longitudinal data, including wearable sensor outputs, electronic health records, and biomarker profiles, to improve temporal prediction, enable continuous monitoring, and facilitate adaptive intervention strategies.

In conclusion, explainable ML using XGBoost can effectively identify adults with T2DM at elevated risk for DEP from a small number of routinely available variables. Both the full and streamlined models outperformed traditional LOG, achieving high discrimination with enhanced efficiency. SHAP analysis yielded transparent, biologically and psychosocially plausible explanations for key risk factors, supporting translation into practical, web-based tools for rapid screening and targeted early intervention to improve mental health outcomes in this vulnerable population.

Supplemental Digital Contents “AI_Report(M20), duplication reprot(M20)” are available for this article (https://links.lww.com/MD/R304, https://links.lww.com/MD/R305).

## Author contributions

**Conceptualization:** Yan Tang, Lei Jia.

**Data curation:** Yan Tang, Lei Jia, Jin Dou.

**Formal analysis:** Yan Tang, Lei Jia, Junjun Zhou.

**Investigation:** Jingjuan Qian.

**Methodology:** Yan Tang, Lei Jia, Junjun Zhou.

**Project administration:** Kim Lam Soh.

**Supervision:** Kim Lam Soh.

**Validation:** Jin Dou.

**Writing – original draft:** Yan Tang, Lei Jia.

**Writing – review & editing:** Xin Yi, Kim Lam Soh.

## Supplementary Material




