# Supplementary material for: Development of an explainable machine learning model for predicting depression in adults with type 2 diabetes mellitus: A cross-sectional SHAP-based analysis of NHANES 2009–2023
Source: Medicine (Baltimore). 2026 Feb 6;105(6):e47522. doi: 10.1097/MD.0000000000047522 (PMC12885714; doi:10.1097/MD.0000000000047522)
Supplement: Supplementary file 2 [file medi-105-e47522-s002.pdf]

M20CCC.docx

作者为 User .

提交日期: 2025年09月27日 09:44上午 (UTC+0530)
提交作业代码: 2763366438
文档名称: M20CCC.docx (45.42K)
文字总数: 6910
字符总数: 44084

Development of an Explainable Machine Learning Model for Predicting Depression in Adults With Type 2 Diabetes Mellitus: A Cross-Sectional SHAP-Based Analysis of NHANES 2009–2023

Abstract

Background: Depression (DEP) is a common yet frequently underdiagnosed comorbidity among individuals with type 2 diabetes mellitus (T2DM). Its presence can aggravate poor glycemic control, accelerate disease progression, and increase the risk of diabetes-related complications. Despite the recognized clinical impact, practical and accurate risk stratification tools that utilize routinely available patient information remain scarce in everyday practice. Improving the identification of high-risk patients through interpretable models may facilitate earlier diagnosis and more effective management. **Methods:** We conducted a cross-sectional analysis using data from adults with T2DM enrolled in the National Health and Nutrition Examination Survey (NHANES) between 2009 and 2023. DEP was classified based on a Patient Health Questionnaire-9 (PHQ-9) score of 10 or higher. Twenty-eight candidate predictors encompassing demographic characteristics, clinical and biochemical measurements, and lifestyle factors were initially included. Variable selection was performed using least absolute shrinkage and selection operator (LASSO) regression. Five machine learning algorithms—Random Forest, Extreme Gradient Boosting (XGBoost), multilayer perceptron, logistic regression, and support vector machine—were trained and evaluated using five-fold cross-validation. The best-performing model was interpreted through SHapley Additive exPlanations (SHAP) analysis to identify the most influential predictors. A streamlined version incorporating the top ten predictors was further developed and implemented as a user-friendly web-based risk estimation tool. **Results:** Among 2,837 participants, 449 (15.8%) were identified as having comorbid DEP. The XGBoost model demonstrated the highest discriminative ability, with a validation area under the receiver operating characteristic curve of 0.888, accuracy of 0.834, F1-score of 0.715, sensitivity of 0.577, and specificity of 0.979, surpassing the performance of the other algorithms evaluated. SHAP analysis revealed gender, poverty-to-income ratio, sleep duration, smoking status, educational levels, race, age, high cholesterol, hypertension, and insulin use as the most influential predictors. A streamlined XGBoost model incorporating only these ten variables achieved an area under the curve of 0.886, closely matching the predictive capability of the full model. The deployed web-based tool enables rapid and individualized estimation of DEP risk in patients with T2DM using routinely available clinical and demographic information. **Conclusions**: Explainable machine learning applied to nationally representative data can accurately identify adults with T2DM at heightened risk of DEP using a small set of non-invasive clinical features. The deployed tool offers a scalable, interpretable, and clinically actionable approach to support early detection and intervention, potentially improving mental health outcomes in this high-risk population.

Keywords: type 2 diabetes mellitus, depression, machine learning, SHapley additive exPlanations, NHANES

1 Introduction

Type 2 diabetes mellitus (T2DM) has become a major global health challenge in the 21st century. [1] According to the International Diabetes Federation (IDF) Diabetes Atlas, approximately 537 million adults aged 20–79 years were living with diabetes in 2021, corresponding to a prevalence of 10.5%. [2,3] Projections indicate that by 2045 the prevalence will increase to 12.2%, with the number of affected individuals expected to reach 783 million—around 46% higher than in 2021. [2,3] Beyond its scale, T2DM

is a leading cause of premature death and disability, contributing to roughly 1.66 million deaths globally in 2021, most attributable to T2DM. [4] The condition is further complicated by chronic complications including cardiovascular disease, [5] chronic kidney disease, [6] retinopathy, [7] and neuropathy, [8] which substantially intensify its impact on patients and healthcare systems.

Depression (DEP) is a common mental health disorder characterized by persistent low mood, anhedonia, and frequent cognitive impairment. [9,10] In 2021, an estimated 330 million people globally were living with DEP [11], which remains a major contributor to non-fatal health loss. [12] The co-occurrence of DEP and T2DM is increasingly recognized as a synergistically harmful combination. [13] Evidence shows that DEP in patients with T2DM accelerates disease progression, [14] raises the risk of microvascular and macrovascular complications, [15] impairs treatment adherence, [16] and exacerbates glycemic dysregulation. These effects heighten healthcare utilization and worsen prognosis, yet many affected individuals remain undiagnosed and undertreated, stressing the need for efficient screening and risk stratification strategies.

Most existing models for predicting DEP risk in T2DM populations rely on conventional regression-based approaches. [17] While valuable, such models often assume linear relationships and may fail to identify complex, non-linear interactions among biological, clinical, and psychosocial predictors. As a result, their predictive performance is frequently modest. Recent advances in machine learning (ML) have shown promise in capturing these intricate patterns, [18] with growing application in clinical practice to enhance risk prediction, improve diagnostic accuracy, and support individualized care. [19] Moreover, interpretability techniques such as SHapley Additive exPlanations (SHAP) allow visualization of the contribution of each predictor to model output, increasing transparency and clinical utility. [20]

In this study, we used data from the National Health and Nutrition Examination Survey (NHANES), a nationally representative dataset comprising standardized demographic, clinical, laboratory, and lifestyle information, to develop an interpretable ML model for identifying DEP risk in adults with T2DM. DEP status was determined using the Patient Health Questionnaire-9 (PHQ-9). [21] SHAP analysis was applied to determine the most influential predictors, from which a simplified, transparent model was created with the aim of enabling earlier detection of high-risk individuals and supporting timely intervention.

2 Methods and Materials

2.1 Study Population

This cross-sectional study analyzed data from the NHANES collected between 2009 and 2023, comprising a total of 83,492 participants. We first excluded individuals without T2DM (n = 77,926) and those with invalid or missing values for key study variables (n = 2,729). After applying these criteria, a total of 2,837 participants with complete data were included in the final analysis. Among them, 449 participants had comorbid DEP (T2DM with DEP group), and 2388 participants did not (T2DM without DEP group). The detailed screening process is shown in **Figure 1**.

2.2 Definition of T2DM and DEP

T2DM was defined based on participants' self-reported medical history, specifically those who answered "Yes" to the question "Have a doctor or health professional ever told you that you have diabetes?", and were aged ≥ 20 years.

Depressive symptoms were evaluated using the Patient Health Questionnaire-9 (PHQ-9), a validated nine-item self-report instrument designed to screen for depressive symptoms over the preceding two weeks. [22] Each item addresses one of the core criteria for major depressive disorder according to the Diagnostic and Statistical Manual of Mental Disorders, Fourth Edition (DSM-IV), [23] including:

1. Little interest or pleasure in doing things?
2. Feeling down, depressed, or hopeless?
3. Trouble sleeping or sleeping too much?
4. Feeling tired or having little energy?
5. Poor appetite or overeating?
6. Feeling bad about oneself?
7. Trouble concentrating on things?
8. Moving or speaking slowly, or being fidgety/restless?
9. Thoughts of self-harm or that one would be better off dead?

Responses for each item were scored from 0 to 3, corresponding to “not at all,” “several days,” “more than half the days,” and “nearly every day,” respectively. Participants endorsing any of the nine symptoms were further asked about the degree to which these problems impaired their daily functioning.

For each participant, a total PHQ-9 score was calculated by summing the item scores. Participants with a total score ≥10 were considered to have clinically significant depressive symptoms. Based on this criterion, the T2DM population was divided into two groups for analysis: T2DM with DEP and T2DM without DEP.

2.3 Clinical Variable Selection and Processing

In this study, we initially considered 32 clinical variables from the NHANES database to capture demographic characteristics, anthropometric measurements, hematologic and biochemical parameters, as well as disease history and lifestyle factors. For categorical variables, records with invalid or missing values were directly excluded. For continuous variables, those with a missing rate ≥20% were removed, while variables with a missing rate ≤20% were subjected to multiple imputation to minimize bias and retain maximal sample information.

After data cleaning and processing, a total of 28 variables were ultimately included in the analysis. These comprised demographic characteristics: Gender, Age, Race, Education, and family income-to-poverty ratio (PIR); anthropometric measurements: Height, Weight, and body mass index (BMI); hematologic and biochemical parameters: high-density lipoprotein cholesterol (HDL-C), white blood cell count (WBC), lymphocyte percentage (Lymph), monocyte percentage (Mono), neutrophil percentage (Neut), eosinophil percentage (Eos), basophil percentage (Baso), red blood cell count (RBC), hemoglobin (HGB), hematocrit (HCT), red cell distribution width (RDW), platelet count (PLT), mean platelet volume (MPV); disease history: Hypertension and .High Cholesterol; diabetes-related medication use: Taking Diabetic Pills and Taking Insulin; and lifestyle factors: sleeping duration per day (Sleep_D) and Smoking status. All variables and their measurement protocols can be accessed on the official NHANES website (https://wwwn.cdc.gov/nchs/nhane)

2.4 ML Model Development and Validation

For model development, 28 clinical variables were initially considered. To reduce potential multicollinearity and prevent overfitting, we applied the Least Absolute Shrinkage and Selection Operator

(LASSO) regression, which performs both variable selection and regularization, retaining only the most informative predictors. Class imbalance in the outcome variable was addressed using the Synthetic Minority Over-sampling Technique (SMOTE), ensuring that the minority class (T2DM with DEP group) was adequately represented in the training set.

In this study, five machine learning algorithms, namely Random Forest (RF), Extreme Gradient Boosting (XGBoost), Support Vector Machine (SVM), Stochastic Gradient Boosting (SMP), and Logistic Regression (LOG), were applied to build predictive models assessing the risk of DEP in patients with T2DM. The complete dataset was randomly divided into a training set comprising 70% of the cases and a validation set containing the remaining 30%. During the model development stage, five-fold cross-validation was conducted within the training set. This procedure enabled refinement of hyperparameters, enhanced the ability of the models to generalize beyond the training data, and reduced the likelihood of overfitting. Model performance was examined using the independent validation set, with evaluation based on multiple indicators including the area under the receiver operating characteristic curve (AUC), accuracy, sensitivity, specificity, and the F1-score, thereby providing a comprehensive assessment of predictive capacity. The algorithm that demonstrated superior and consistent results across all evaluation metrics was identified as the optimal model and reserved for subsequent detailed analysis.

2.5 Model Simplification, Interpretation, and Deployment

To improve the interpretability of the selected machine learning model and to support its potential use in clinical settings, SHapley Additive exPlanations (SHAP) analysis was conducted. The SHAP importance values for each variable were calculated to quantify their relative contributions to the model's predictions. Variables were ordered according to these importance scores, and the ten predictors with the highest contributions were identified as the most influential factors. Following this step, SHAP dependence plots were examined to characterize both the direction and the magnitude of each selected variable's association with the predicted risk of DEP in patients with T2DM. This analysis provided a visual and quantitative understanding of how changes in individual features impact the likelihood of comorbid DEP.

Using the ten most influential predictors, a streamlined XGBoost model was developed. Performance evaluation for this simplified model was carried out using the same metrics applied to the original analysis: area under the AUC, accuracy, sensitivity, specificity, and the F1-score. The results indicated that, despite the reduction in the number of input features, the simplified model maintained a high level of predictive accuracy. This finding suggests that the most important risk factors identified by SHAP accounted for the majority of the predictive information present in the full set of variables.

Finally, this simplified model was deployed as a web-based interactive application, enabling clinicians to input patient-specific information and obtain individualized risk predictions for comorbid DEP in T2DM patients. This tool provides a user-friendly and clinically actionable platform to support decision-making in routine practice.

2.6 Statistical Analysis

All analysis were performed using R software (version 4.5.0). Continuous variables were first assessed for normality using the Kolmogorov–Smirnov test and skewness. Variables showing no significant deviation from normality ($P > 0.05$) or with skewness ≤ 1 were summarized as mean ± standard deviation (SD), whereas those with significant deviation from normality and skewness > 1 were summarized as median with interquartile range (IQR). A p value < 0.05 was considered statistically significant.

3 Methods and Materials

3.1 Baseline Characteristics of T2DM Participants with and without DEP

A total of 2,837 participants with T2DM were included, of whom 449 had comorbid DEP. Participants with DEP were younger and had lower PIR, higher BMI and weight, and slightly shorter height compared with those without DEP (all P < 0.05). Most laboratory measurements, including HDL, Lymph, Neut, Mono, Eos, Baso, and Glu, were similar between groups. Participants with DEP showed modestly higher WBC, HGB, HCT, RDW, PLT, and MPV compared with non-depressed participants (all P < 0.05), whereas daily sleep duration was slightly shorter (P = 0.022). In categorical variables, DEP was more prevalent among females, while males were less represented in the DEP group (P < 0.001). Race/ethnicity distributions differed slightly between groups (P = 0.017), with higher proportions of Mexican American and Other Hispanic participants and slightly lower proportions of Non-Hispanic Black and Other Race participants in the DEP group. Participants with DEP were more likely to have lower educational attainment, including <9th grade or 9–11th grade, and were less likely to be college graduates (P < 0.001). Regarding comorbidities, hypertension and high cholesterol were more common in participants with DEP (both P = 0.003), whereas insulin use was slightly higher (P = 0.026) and oral diabetic medication use did not differ significantly (P = 0.120). Smoking prevalence was higher in the DEP group (P = 0.003). As shown in **Table 1**.

3.2 Comparison of ML Models and Selection of the Optimal Predictor

We systematically compared the predictive performance of five machine learning algorithms—RF, XGBoost, MLP, LOG, and SVM—for DEP in patients with T2DM. In the training set, XGBoost exhibited the highest discriminative ability, achieving an AUC of 0.912 (95% CI 0.900–0.924), with a balanced overall performance: accuracy 0.852, F1-score 0.746, sensitivity 0.603, and specificity 0.992. RF and MLP also demonstrated substantial predictive capability, though slightly lower than XGBoost (RF AUC 0.895, MLP AUC 0.856), while LOG and SVM showed moderate discrimination (AUCs 0.728 and 0.737, respectively), indicating limited ability to identify DEP cases in this cohort. In the validation set, XGBoost maintained robust performance, with an AUC of 0.888 (95% CI 0.867–0.909), accuracy 0.834, F1-score 0.715, sensitivity 0.577, and specificity 0.979, demonstrating stable generalization. RF showed slightly lower performance compared with XGBoost, whereas LOG and SVM exhibited comparatively modest discriminative ability.

Collectively, these results highlight the superiority of ensemble tree-based methods for DEP risk stratification in T2DM, with XGBoost demonstrating the optimal combination of discrimination, calibration, and robustness. Its superior performance can be attributed to its ability to capture complex non-linear relationships, handle feature interactions effectively, and maintain stability across training and validation sets. Therefore, XGBoost was selected as the optimal predictive model for subsequent analysis. As shown in **Figure 2**.

3.3 SHAP Analysis of the Optimal XGBoost Model

To further elucidate the contribution of individual features to the prediction of comorbid DEP in patients with T2DM, we applied SHAP to the optimal XGBoost model. Initially, variable importance was ranked according to the mean absolute SHAP values, and the top 10 predictors were selected for detailed interpretation using SHAP dependence plots, as shown in **Figure 3A**. The top-ranked features, in descending order of importance, were:Gender (0.242), PIR (0.157), SLP_D (0.147), Smoking (0.103),

Education (0.098), Race (0.092), Age (0.089), High cholesterol (0.087), Hypertension (0.078), and Taking insulin (0.038).

For PIR, higher values were associated with progressively lower SHAP values, indicating a shift in the model prediction towards the non-depressed state and suggesting a reduced risk of DEP with increasing PIR. Regarding Age, SHAP values remained relatively high and stable before approximately 62 years, corresponding to an elevated predicted risk of DEP; beyond this threshold, a sharp decline in SHAP values was observed, indicating a pronounced reduction in DEP risk in older individuals. SLP_D demonstrated a non-linear relationship with DEP risk: values <6 h were associated with the highest SHAP values and, consequently, the greatest predicted risk. Between 6–8 h, SHAP values markedly declined, indicating a protective effect of adequate sleep, whereas durations ≥9–10 h were again linked with elevated SHAP values, reflecting increased risk. Moreover, our streamlined model, which includes only ten variables, retained substantial discriminative performance, highlighting the efficiency of SHAP-guided feature selection and demonstrating the model's readiness for real-time, low-burden clinical implementation.

Regarding Education, participants with less than 9th grade and 9–11th grade education exhibited higher SHAP values, indicating the highest predicted risk of DEP among T2DM patients. In contrast, as educational attainment increased, DEP risk progressively declined, reaching the lowest predicted risk among those with a college graduate. Concerning Race, Non-Hispanic White participants had the highest SHAP values, whereas Mexican American and Other Hispanic individuals showed slightly lower but still relatively high SHAP values, suggesting elevated DEP risk in these three groups. By contrast, Non-Hispanic Black and Other Race participants had low SHAP values, indicating a lower predicted risk of DEP. For treatment-related variables, Taking Insulin demonstrated a complex relationship with predicted DEP risk in the SHAP analysis. Patients who answered "Yes" exhibited predominantly moderate-to-high SHAP values, suggesting that insulin use was associated with an elevated predicted risk of comorbid DEP. In contrast, those who responded "No" generally showed low-to-moderate SHAP values, indicating a lower predicted risk among non-insulin users. Notably, there was partial overlap in SHAP values between the two groups, highlighting that the effect of insulin use on DEP risk may be context-dependent and potentially bidirectional under different clinical conditions. In addition, "Yes" responses for Smoking, Hypertension, and High Cholesterol were consistently linked with higher SHAP values, indicating an increased likelihood of comorbid DEP in these patients. Regarding Gender, female participants exhibited notably higher SHAP values compared to males, suggesting a substantially greater risk of DEP among women with T2DM, as shown in **Figure 3B**.

3.4 XGBoost Model Simplification and Deployment

To enhance applicability in clinical practice, we retrained a streamlined XGBoost model that incorporated only the top 10 features identified by SHAP analysis. This reduction in input variables offers several advantages: it lowers the burden of data collection, shortens computation time, improves interpretability, and facilitates integration into real-time clinical workflows.

On the training set, the simplified model achieved an AUC of 0.908 (95% CI: 0.896–0.921), accuracy of 0.854, sensitivity of 0.609, specificity of 0.992, and an F1-score of 0.750. Performance on the validation set was comparably strong, with an AUC of 0.886 (95% CI: 0.865–0.908), accuracy of 0.833, sensitivity of 0.577, specificity of 0.978, and an F1-score of 0.714. These metrics were closely aligned with those of the full-variable XGBoost model (validation AUC = 0.888, accuracy = 0.834, sensitivity = 0.577, specificity = 0.979, F1-score = 0.715), indicating that predictive power was largely preserved despite the reduction in model complexity. As shown in **Figure 4**.

Given its robust performance, the simplified model was subsequently deployed as a web-based application. This tool enables clinicians to input a limited set of clinical and demographic variables and obtain an individualized estimate of high DEP risk among patients with T2DM, supporting early identification and timely intervention. As shown in **Figure 5**.

4 Discussion

This study developed and validated an explainable ML approach to predict DEP risk in adults with T2DM, achieving high discrimination while retaining interpretability through SHAP analysis. Among the five ML algorithms evaluated, XGBoost provided the best overall performance, and a streamlined variant using only ten top-ranked predictors achieved nearly identical results, demonstrating the feasibility of a clinically efficient model.

When compared with prior work [17] based on multiple logistic regression, both our full-variable and streamlined XGBoost models demonstrated superior predictive performance for DEP risk in adults with T2DM across all major evaluation metrics. In the referenced regression study, the training set achieved a sensitivity of 62.4%, specificity of 80.0%, accuracy of 77.6%, and an AUC of 0.780, while the testing set yielded 65.6% sensitivity, 75.4% specificity, 74.2% accuracy, and an AUC of 0.752. In contrast, our full-variable XGBoost model reached, in the training set, an AUC of 0.912, accuracy of 85.2%, sensitivity of 60.3%, specificity of 99.2%, and F1-score of 0.746; and in the validation set, an AUC of 0.888, accuracy of 83.4%, sensitivity of 57.7%, specificity of 97.9%, and F1-score of 0.715. The streamlined ten-predictor XGBoost performed almost identically, achieving a training set AUC of 0.908, accuracy of 85.4%, sensitivity of 60.9%, specificity of 99.2%, and F1-score of 0.750; and a validation set AUC of 0.886, accuracy of 83.3%, sensitivity of 57.7%, specificity of 97.8%, and F1-score of 0.714. Notably, while sensitivity values across our models were on par with the logistic regression benchmark, specificity and AUC were consistently and substantially higher, indicating more accurate separation of depressed and non-depressed cases. Moreover, our streamlined model, which includes only ten variables, retained substantial discriminative performance, highlighting the efficiency of SHAP-guided feature selection and demonstrating the model' s readiness for real-time, low-burden clinical implementation.

SHAP analysis identified female sex, poverty-to-income ratio, sleep duration, smoking status, educational attainment, race, age, hypercholesterolemia, hypertension, and insulin use as the most influential predictors of DEP in T2DM. Several of these overlapped with variables included in prior multiple logistic regression analysis [18], namely age, gender, PIR, BMI, educational attainment, smoking status, LDL-C, sleep duration, and sleep disorder, indicating a degree of consistency across methods, yet with notable differences in both variable inclusion and relationship patterns.

In patients with T2DM, the risk of DEP appears to be closely associated with demographic factors, particularly sex and age. Female patients showed a higher likelihood of depressive symptoms, possibly due to sex hormone–mediated regulation of neurochemical and immune pathways. Estrogen plays a critical role in enhancing central serotonergic and dopaminergic neurotransmission and promoting neurogenesis; its decline during perimenopause and postmenopause can reduce serotonin synthesis and receptor sensitivity, impairing mood regulation. [24, 25] Moreover, estrogen deficiency promotes the production of proinflammatory cytokines such as interleukin-6 (IL-6) and tumor necrosis factor-α (TNF-α), which contribute to neuroinflammation, hippocampal dysfunction, and may exacerbate insulin resistance and depressive symptoms. [26, 27] Regarding age, the highest risk was noted in individuals aged 20–62 years, consistent with previous evidence that earlier or middle-age onset of T2DM is more strongly

linked to the incidence and severity of depressive symptoms. [28] Patients in this age range often face substantial occupational and family responsibilities, and the long-term burden of disease management heightens psychological stress. [29] diagnosis at this stage also implies longer disease duration and greater cumulative risk of complications, triggering anxiety and DEP. [30] Furthermore, persistent reductions in quality of life and self-efficacy serve as important psychological mechanisms underlying DEP in these individuals. [31, 32] By contrast, in older age, the alleviation of work-related stress and expansion of social networks may partially offset depressive risk.

Socio-cultural factors also emerged as significant determinants of DEP risk in individuals with T2DM. Socioeconomic disadvantage, in particular, exerts its influence through both neuroendocrine and psychosocial pathways. Chronic financial strain and social adversity have been associated with dysregulation of the hypothalamic–pituitary–adrenal (HPA) axis and abnormal cortisol dynamics, which may impair hippocampal neuroplasticity and disrupt glucocorticoid feedback sensitivity. [33, 34] In T2DM, such HPA alterations can aggravate insulin resistance and inflammatory activity, further linking metabolic disturbances to mood disorders. [35] Psychosocial mechanisms, including restricted access to stable housing, nutritious food, healthcare, and supportive social networks, impose sustained stress and increase psychological burden, thereby amplifying vulnerability to DEP. [36, 37] Consistent with previous reports, [38] our findings indicate that patients with lower educational attainment are at higher risk, possibly due to reduced health literacy and diminished capacity to understand and implement effective self-management strategies, [39] leading to suboptimal glycemic control, more frequent complications, and heightened psychological distress. [40, 41] Ethnic differences were also observed: individuals identified as "Other Hispanic" and "Non-Hispanic White" had the highest DEP prevalence, with Mexican Americans also showing elevated rates. These disparities may reflect the combined impact of socioeconomic position, inequitable access to healthcare, and variations in cultural norms and social support resources. [42]

Lifestyle factors were also significantly associated with DEP risk in patients with T2DM. Sleep duration demonstrated a U-shaped relationship with depressive symptoms, whereby both insufficient and prolonged sleep were linked to greater risk. Short sleep may disrupt circadian rhythms, heighten sympathetic nervous system activity, elevate nocturnal cortisol secretion, and reduce slow-wave sleep, ultimately impairing glucose homeostasis and emotional regulation. [43, 44] Conversely, excessive sleep has also been associated with increased DEP risk, although the magnitude of risk appears lower than that observed with sleep deficiency. [45, 46] In contrast, maintaining approximately six to eight hours of sleep per night seems to favor circadian rhythm alignment, a balanced architecture of rapid eye movement and slow-wave sleep, and stable neuroendocrine function, thereby enhancing resilience to depressive symptoms. In our study, smoking was likewise associated with higher DEP risk among individuals with T2DM. Mechanistically, nicotine may acutely stimulate dopamine, serotonin, and norepinephrine release, transiently elevating mood, but chronic exposure results in receptor down-regulation and neurotransmitter depletion, thereby increasing vulnerability to mood disorders. [47, 48] Moreover, smoking in T2DM accelerates endothelial dysfunction and oxidative stress, further contributing to depressive risk. [49, 50] Elevated inflammatory markers have also been observed in patients with comorbid T2DM and DEP, [51] and given that smoking is often accompanied by unhealthy lifestyle behaviors and poor glycemic control, these factors may synergistically amplify psychological burden and depressive susceptibility. [52] We also found that patients with T2DM receiving insulin therapy exhibited an overall higher risk of DEP, consistent with findings from previous studies. [53] This association may be partly explained by the fact that insulin users typically have longer disease duration, more advanced disease severity, and heavier psychological burden. [54] In addition, the demands of intensive blood glucose monitoring and injection management may further increase treatment-related stress. Interestingly, overlapping SHAP values between insulin users and non-users suggest a bidirectional effect: in certain populations, insulin therapy

may improve glycemic control, stabilize disease progression, and thereby mitigate depressive symptoms. [15]

In patients with T2DM, the presence of hypercholesterolemia or hypertension is associated with a markedly elevated risk of DEP. These vascular–metabolic risk factors may contribute to depressive vulnerability by distinct but converging mechanisms: hypercholesterolemia promotes atherosclerotic processes, whereas hypertension accelerates microvascular injury. Both pathways impair cerebral blood flow regulation and neurovascular coupling, thereby reducing metabolic support to the prefrontal cortex and limbic circuits that are critically involved in mood regulation. [55, 56] Moreover, the cumulative burden of multiple chronic comorbidities represents an additional psychosocial risk factor for DEP. As the number of coexisting conditions increases, patients often experience heightened daily stress and treatment demands, which further amplify the likelihood of depressive symptoms. [57]

By incorporating biological, behavioral, socioeconomic, and treatment-related factors into an interpretable machine learning framework, this study demonstrates that explainable ML can achieve predictive accuracy comparable to—and in certain aspects exceeding—that of conventional epidemiological models. Using a concise set of routinely collected, non-invasive variables, our approach enabled high-performance, individualized risk stratification for DEP in adults with T2DM. The model integrates sex hormone–related neurobiological mechanisms, lifestyle patterns, social determinants, and treatment burden into a transparent structure, providing clinically meaningful and actionable insights.

Several limitations should be noted. First, the cross-sectional design limits causal inference; longitudinal research is required to clarify temporal dynamics and potential bidirectional associations between diabetes, its determinants, and DEP. Second, DEP status was based on the PHQ-9 self-report instrument rather than structured clinical interviews, introducing possible misclassification despite its broad validation in epidemiological studies. Third, while internal validation indicates robustness, external validation in independent, prospective, multicenter cohorts is necessary to verify generalizability across diverse healthcare systems, cultural contexts, and demographic groups. Additionally, psychosocial factors such as life events, coping styles, and social support were not available in the NHANES dataset but may further refine model performance. Future work should explore the integration of multi-source longitudinal data, including wearable sensor outputs, electronic health records, and biomarker profiles, to improve temporal prediction, enable continuous monitoring, and facilitate adaptive intervention strategies.

In conclusion, explainable machine learning using XGBoost can effectively identify adults with T2DM at elevated risk for DEP from a small number of routinely available variables. Both the full and streamlined models outperformed traditional logistic regression, achieving high discrimination with enhanced efficiency. SHAP analysis yielded transparent, biologically and psychosocially plausible explanations for key risk factors, supporting translation into practical, web-based tools for rapid screening and targeted early intervention to improve mental health outcomes in this vulnerable population.

8 References

[1] He, K. J., Wang, H., Xu, J., Gong, G., Liu, X., & Guan, H. (2024). Global burden of type 2 diabetes mellitus from 1990 to 2021, with projections of prevalence to 2044: A systematic analysis across SDI levels for the global burden of disease study 2021. *Frontiers in Endocrinology*, *15*, 1501690. https://doi.org/10.3389/fendo.2024.1501690. PMID: 39583961; PMCID: PMC11581865

[2] Sun, H., Saeedi, P., Karuranga, S., Pinkepank, M., Ogurtsova, K., Duncan, B. B., … Bommer, C. (2022). IDF Diabetes Atlas: Global, regional and country-level diabetes prevalence estimates for 2021 and projections for 2045. *Diabetes Research and Clinical Practice*, *183*, 109119. https://doi.org/10.1016/j.diabres.2021.109119. PMID: 34879977; PMCID: PMC11057359

[3] Kumar, A., Gangwar, R., Zargar, A. A., Kumar, R., & Sharma, A. (2024). Prevalence of diabetes in India: A review of IDF Diabetes Atlas 10th Edition. *Current Diabetes Reviews*, *20*(1), e130423215752. https://doi.org/10.2174/1573399819666230413094200. PMID: 37069712

[4] Pan, C., Cao, B., Fang, H., Liu, Y., Zhang, S., Luo, W., & Wu, Y. (2025). Global burden of diabetes mellitus 1990–2021: Epidemiological trends, geospatial disparities, and risk factor dynamics. *Frontiers in Endocrinology*, *16*, 1596127. https://doi.org/10.3389/fendo.2025.1596127. PMID: 40666058; PMCID: PMC12259457

[5] Yun, J. S., & Ko, S. H. (2021). Current trends in epidemiology of cardiovascular disease and cardiovascular risk management in type 2 diabetes. *Metabolism*, *123*, 154838. https://doi.org/10.1016/j.metabol.2021.154838. PMID: 34333002

[6] He, Y., Wang, X., Li, L., Liu, M., Wu, Y., Chen, R., … Li, X. (2025). Global, regional, and national prevalence of chronic type 2 diabetic kidney disease from 1990 to 2021: A trend and health inequality analyses based on the Global Burden of Disease Study 2021. *Journal of Diabetes*, *17*(5), e70098. https://doi.org/10.1111/1753-0407.70098. PMID: 40400440; PMCID: PMC12096015

[7] Wong, T. Y., & Tan, T. E. (2023). The diabetic retinopathy "pandemic" and evolving global strategies: The 2023 Friedenwald Lecture. *Investigative Ophthalmology & Visual Science*, *64*(15), 47. https://doi.org/10.1167/iovs.64.15.47. PMID: 38153754; PMCID: PMC10756246

[8] Savelieff, M. G., Elafros, M. A., Viswanathan, V., Jensen, T. S., Bennett, D. L., & Feldman, E. L. (2025). The global and regional burden of diabetic peripheral neuropathy. *Nature Reviews Neurology*, *21*(1), 17–31. https://doi.org/10.1038/s41582-024-01041-y. PMID: 39639140

[9] Malhi, G. S., & Mann, J. J. (2018). Depression. *The Lancet*, *392*(10161), 2299–2312. https://doi.org/10.1016/S0140-6736(18)31948-2. PMID: 30396512

[10] Friedrich, M. J. (2017). Depression is the leading cause of disability around the world. *JAMA*, *317*(15), 1517. https://doi.org/10.1001/jama.2017.3826. PMID: 28418490

[11] Rong, J., Wang, X., Cheng, P., Li, D., & Zhao, D. (2025). Global, regional and national burden of depressive disorders and attributable risk factors, from 1990 to 2021: Results from the 2021 Global Burden of Disease study. *British Journal of Psychiatry*. Advance online publication. https://doi.org/10.1192/bjp.2024.266. PMID: 39809717

[12] Liu, J., Ye, Z., Cai, Y., Li, J., Dong, Z., Zhang, X., & Lei, M. (2025). Unmasking the rising global burden of depression: A 32-year GBD analysis of gender disparities and regional hotspots in Sub-Saharan Africa. *PLoS ONE*, *20*(7), e0326974. https://doi.org/10.1371/journal.pone.0326974. PMID: 40743274; PMCID: PMC12312894

[13] Yoshimura, R., & Watanabe, C. (2025). Comorbidity of major depression and type 2 diabetes mellitus: Epidemiology, pathophysiology, and treatment approaches. *Journal of UOEH*, *47*(2), 95–103. https://doi.org/10.7888/juoeh.47.95. PMID: 40451800

[14] Xu, H., & Chen, Q. (2025). The bidirectional influence between type 2 diabetes mellitus and the state of depression and anxiety. *Journal of Affective Disorders*, *386*, 119467. https://doi.org/10.1016/j.jad.2025.119467. PMID: 40419162

[15] Fanelli, G., Raschi, E., Hafez, G., Matura, S., Schiweck, C., Poluzzi, E., & Lunghi, C. (2025). The interface of depression and diabetes: Treatment considerations. *Translational Psychiatry*, *15*(1), 22. https://doi.org/10.1038/s41398-025-03234-5. PMID: 39856085; PMCID: PMC11760355

[16] Yang, H., Wu, F., Gui, M., Cheng, Y., & Zhang, L. (2023). The role of medication adherence in the association between depressive symptoms and quality of life in older adults with type 2 diabetes mellitus. *BMC Geriatrics*, *23*(1), 196. https://doi.org/10.1186/s12877-023-03929-8. PMID: 36997851; PMCID: PMC10064516

[17] Yu, X., Tian, S., Wu, L., Zheng, H., Liu, M., & Wu, W. (2024). Construction of a depression risk prediction model for type 2 diabetes mellitus patients based on NHANES 2007–2014. *Journal of Affective Disorders*, *349*, 217–225. https://doi.org/10.1016/j.jad.2024.01.083. PMID: 38199400

[18] Alhumaidi, N. H., Dermawan, D., & Kamaruzaman, H. F., Alotaiq, N. (2025). The use of machine learning for analyzing real-world data in disease prediction and management: Systematic review. *JMIR Medical Informatics*, *13*, e68898. https://doi.org/10.2196/68898. PMID: 40537090; PMCID: PMC12226786

[19] Tuppad, A., & Patil, S. D. (2022). Machine learning for diabetes clinical decision support: A review. *Advances in Computational Intelligence*, *2*(2), 22. https://doi.org/10.1007/s43674-022-00034-y. PMID: 35434723; PMCID: PMC9006199

[20] Alnazari, N., Alanazi, O. I., Alosaimi, M. O., Alanazi, Z. M., Alhajeri, Z. M., Alhussaini, K. M., … Azzam, A. Y. (2025). Development of explainable artificial intelligence based machine learning model for predicting 30-day hospital readmission after renal transplantation. *BMC Nephrology*, *26*(1), 203. https://doi.org/10.1186/s12882-025-04128-w. PMID: 40264055; PMCID: PMC12013099

[21] Janssen, E. P., Köhler, S., Stehouwer, C. D., Schaper, N. C., Dagnelie, P. C., Sep, S. J., … Schram, M. T. (2016). The Patient Health Questionnaire-9 as a screening tool for depression in individuals with type 2 diabetes mellitus: The Maastricht Study. *Journal of the American Geriatrics Society*, *64*(11), e201–e206. https://doi.org/10.1111/jgs.14388. PMID: 27783384

[22] Kroenke, K., Spitzer, R. L., & Williams, J. B. (2001). The PHQ-9: Validity of a brief depression severity measure. *Journal of General Internal Medicine*, *16*(9), 606–613. https://doi.org/10.1046/j.1525-1497.2001.016009606.x. PMID: 11556941; PMCID: PMC1495268

[23] Spitzer, R. L., Kroenke, K., & Williams, J. B. (1999). Validation and utility of a self-report version of PRIME-MD: The PHQ primary care study. *JAMA*, *282*(18), 1737–1744. https://doi.org/10.1001/jama.282.18.1737. PMID: 10568646

[24] Gagne, C., Piot, A., & Brake, W. G. (2022). Depression, estrogens, and neuroinflammation: A preclinical review of ketamine treatment for mood disorders in women. *Frontiers in Psychiatry*, *12*, 797577. https://doi.org/10.3389/fpsyt.2021.797577. PMID: 35115970; PMCID: PMC8804176

[25] Albert, K. M., & Newhouse, P. A. (2019). Estrogen, stress, and depression: Cognitive and biological interactions. *Annual Review of Clinical Psychology*, *15*, 399–423. https://doi.org/10.1146/annurev-clinpsy-050718-095557. PMID: 30786242; PMCID: PMC9673602

[26] Zhang, Y., Tan, X., & Tang, C. (2024). Estrogen-immuno-neuromodulation disorders in menopausal depression. *Journal of Neuroinflammation*, *21*(1), 159. https://doi.org/10.1186/s12974-024-03152-1. PMID: 38898454; PMCID: PMC11188190

[27] Xiang, X., Palasuberniam, P., & Pare, R. (2024). The role of estrogen across multiple disease mechanisms. *Current Issues in Molecular Biology*, *46*(8), 8170–8196. https://doi.org/10.3390/cimb46080483. PMID: 39194700; PMCID: PMC11352819

[28] Barker, M. M., Davies, M. J., Zaccardi, F., Brady, E. M., Hall, A. P., Henson, J. J., … Yates, T. (2023). Age at diagnosis of type 2 diabetes and depressive symptoms, diabetes-specific distress, and self-compassion. *Diabetes Care*, *46*(3), 579–586. https://doi.org/10.2337/dc22-1237. PMID: 36630531; PMCID: PMC10020022

[29] Morales-Brown, L. A., Perez Algorta, G., & Salifu, Y. (2024). Understanding experiences of diabetes distress: A systematic review and thematic synthesis. *Journal of Diabetes Research*, *2024*, 3946553. https://doi.org/10.1155/2024/3946553. PMID: 39574786; PMCID: PMC11581805

[30] Strandberg, R. B., Nilsen, R. M., Pouwer, F., Igland, J., Røssberg, J. I., Jenum, A. K., … Iversen, M. M. (2025). Pharmacologically treated depression, anxiety, and insomnia in individuals with type 2 diabetes: The role of diabetes duration, age, and age at diabetes onset. A Norwegian population-based registry study from the OMIT cohort. *Journal of Psychosomatic Research*, *190*, 112057. https://doi.org/10.1016/j.jpsychores.2025.112057. PMID: 39955944

[31] Bayani, M. A., Shakiba, N., Bijani, A., & Moudi, S. (2022). Depression and quality of life in patients with type 2 diabetes mellitus. *Caspian Journal of Internal Medicine*, *13*(2), 335–342. https://doi.org/10.22088/cjim.13.2.3. PMID: 35919653; PMCID: PMC9301220

[32] Ibrahim, N. F., Nofal, H. A., Ali, H. T., El-Rafey, D. S., Almadani, N., Mahfouz, R., … Khodary, R. M. (2025). Enhancing self-care management in diabetic patients: A randomized controlled trial exploring the interplay of social support, self-efficacy, and empowerment. *Acta Diabetologica*. Advance online publication. https://doi.org/10.1007/s00592-025-02498-z. PMID: 40237832

[33] Merz, E. C., Myers, B., Hansen, M., Simon, K. R., Strack, J., & Noble, K. G. (2023). Socioeconomic disparities in hypothalamic-pituitary-adrenal axis regulation and prefrontal cortical structure. *Biological Psychiatry Global Open Science*, *4*(1), 83–96. https://doi.org/10.1016/j.bpsgos.2023.10.004. PMID: 38090738; PMCID: PMC10714216

[34] Dowd, J. B., Simanek, A. M., & Aiello, A. E. (2009). Socio-economic status, cortisol and allostatic load: A review of the literature. *International Journal of Epidemiology*, *38*(5), 1297–1309. https://doi.org/10.1093/ije/dyp277. PMID: 19720725; PMCID: PMC2755130

[35] Diz-Chaves, Y., Gil-Lozano, M., Toba, L., Fandiño, J., Ogando, H., González-Matías, L. C., & Mallo, F. (2016). Stressing diabetes? The hidden links between insulinotropic peptides and the HPA axis. *Journal of Endocrinology*, *230*(2), R77–R94. https://doi.org/10.1530/JOE-16-0118. PMID: 27325244

[36] Lund, C., Brooke-Sumner, C., Baingana, F., Baron, E. C., Breuer, E., Chandra, P., … Patel, V. (2018). Social determinants of mental disorders and the Sustainable Development Goals: A systematic review of reviews. *Lancet Psychiatry*, *5*(4), 357–369. https://doi.org/10.1016/S2215-0366(18)30060-9. PMID: 29580610

[37] Bharmal, N. (2022). Social determinants and health equity in functional medicine. *Physical Medicine and Rehabilitation Clinics of North America*, *33*(3), 665–678. https://doi.org/10.1016/j.pmr.2022.04.007. PMID: 35989057

[38] Liu, X., Li, Y., Guan, L., He, X., Zhang, H., Zhang, J., … Jin, R. (2022). A systematic review and meta-analysis of the prevalence and risk factors of depression in type 2 diabetes patients in China. *Frontiers in Medicine*, *9*, 759499. https://doi.org/10.3389/fmed.2022.759499. PMID: 35620713; PMCID: PMC9127805

[39] Kim, E. (2024). Exploring the relationship between health literacy and depression among individuals with chronic conditions: Insights from the KCHS. *Healthcare*, *12*(19), 1927. https://doi.org/10.3390/healthcare12191927. PMID: 39408107; PMCID: PMC11476441

[40] Maleki Chollou, K., Gaffari-Fam, S., Babazadeh, T., Daemi, A., Bahadori, A., & Heidari, S. (2020). The association of health literacy level with self-care behaviors and glycemic control in a low education population with type 2 diabetes mellitus: A cross-sectional study in Iran. *Diabetes, Metabolic Syndrome and Obesity*, *13*, 1685–1693. https://doi.org/10.2147/DMSO.S253607. PMID: 32547130; PMCID: PMC7245439

[41] Ong-Artborirak, P., Seangpraw, K., Boonyathee, S., Auttama, N., & Winaiprasert, P. (2023). Health literacy, self-efficacy, self-care behaviors, and glycemic control among older adults with type 2 diabetes mellitus: A cross-sectional study in Thai communities. *BMC Geriatrics*, *23*(1), 297. https://doi.org/10.1186/s12877-023-04010-0. PMID: 37193967; PMCID: PMC10185940

[42] Bailey, R. K., Mokonogho, J., & Kumar, A. (2019). Racial and ethnic differences in depression: Current perspectives. *Neuropsychiatric Disease and Treatment*, *15*, 603–609. https://doi.org/10.2147/NDT.S128584. PMID: 30863081; PMCID: PMC6390869

[43] Morris, C. J., Aeschbach, D., & Scheer, F. A. (2012). Circadian system, sleep and endocrinology. *Molecular and Cellular Endocrinology*, *349*(1), 91–104. https://doi.org/10.1016/j.mce.2011.09.003. PMID: 21939733; PMCID: PMC3242827

[44] Lee, S. W. H., Ng, K. Y., & Chin, W. K. (2017). The impact of sleep amount and sleep quality on glycemic control in type 2 diabetes: A systematic review and meta-analysis. *Sleep Medicine Reviews*, *31*, 91–101. https://doi.org/10.1016/j.smrv.2016.02.001. PMID: 26944909

[45] Dong, L., Xie, Y., & Zou, X. (2022). Association between sleep duration and depression in US adults: A cross-sectional study. *Journal of Affective Disorders*, *296*, 183–188. https://doi.org/10.1016/j.jad.2021.09.075. PMID: 34607059

[46] Itani, O., Jike, M., Watanabe, N., & Kaneita, Y. (2017). Short sleep duration and health outcomes: A systematic review, meta-analysis, and meta-regression. *Sleep Medicine*, *32*, 246–256. https://doi.org/10.1016/j.sleep.2016.08.006. PMID: 27743803

[47] Picciotto, M. R., Lewis, A. S., van Schalkwyk, G. I., & Mineur, Y. S. (2015). Mood and anxiety regulation by nicotinic acetylcholine receptors: A potential pathway to modulate aggression and related behavioral states. *Neuropharmacology*, *96*(Pt B), 235–243. https://doi.org/10.1016/j.neuropharm.2014.12.028. PMID: 25582289; PMCID: PMC4486625

[48] Mineur, Y. S., & Picciotto, M. R. (2010). Nicotine receptors and depression: Revisiting and revising the cholinergic hypothesis. *Trends in Pharmacological Sciences*, *31*(12), 580–586. https://doi.org/10.1016/j.tips.2010.09.004. PMID: 20965579; PMCID: PMC2991594

[49] Pan, A., Keum, N., Okereke, O. I., Sun, Q., Kivimaki, M., Rubin, R. R., & Hu, F. B. (2012). Bidirectional association between depression and metabolic syndrome: A systematic review and meta-analysis of epidemiological studies. *Diabetes Care*, *35*(5), 1171–1180. https://doi.org/10.2337/dc11-2055. PMID: 22517938; PMCID: PMC3329841

[50] Hackett, R. A., & Steptoe, A. (2017). Type 2 diabetes mellitus and psychological stress – a modifiable risk factor. *Nature Reviews Endocrinology*, *13*(9), 547–560. https://doi.org/10.1038/nrendo.2017.64. PMID: 28664919

[51] Nguyen, M. M., Perlman, G., Kim, N., Wu, C. Y., Daher, V., Zhou, A., … Swardfager, W. (2021). Depression in type 2 diabetes: A systematic review and meta-analysis of blood inflammatory markers. *Psychoneuroendocrinology*, *134*, 105448. https://doi.org/10.1016/j.psyneuen.2021.105448. PMID: 34687965

[52] Sia, H. K., Kor, C. T., Tu, S. T., Liao, P. Y., & Wang, J. Y. (2022). Association between smoking and glycemic control in men with newly diagnosed type 2 diabetes: A retrospective matched cohort study. *Annals of Medicine*, *54*(1), 1381–1393. https://doi.org/10.1080/07853890.2022.2098081. PMID: 35978604

[53] Gumuskaya, P. O., Altun, O., Yildirim, E., Yuztas, N. K., Ozsoy, N., Kalyon, S., Irak, L., Ozcan, M., Altun, Z. O., Demir, P. S., Cil, E. O., Arman, Y., Uzun, H., & Tukek, T. (2025). The Association Between Depression and Antidiabetic Treatments in Type 2 Diabetes Patients with Both Good and Poor Glycemic Control. Journal of clinical medicine, 14(10), 3460. https://doi.org/10.3390/jcm14103460

[54] Bai, X., Liu, Z., Li, Z., & Yan, D. (2018). The association between insulin therapy and depression in patients with type 2 diabetes mellitus: a meta-analysis. BMJ open, 8(11), e020062. https://doi.org/10.1136/bmjopen-2017-020062

[55] Luchsinger, J. A., Tang, M. X., Shea, S., & Mayeux, R. (2001). Hyperinsulinemia and risk of Alzheimer disease. *Neurology*, *57*(8), 1471–1478. https://doi.org/10.1212/wnl.57.8.1471. PMID: 11571146

[56] Esposito, K., Chiodini, P., Capuano, A., Bellastella, G., Maiorino, M. I., & Giugliano, D. (2012). Metabolic syndrome and risk of cancer: A systematic review and meta-analysis. *Diabetes Care*, *35*(11), 2402–2411. https://doi.org/10.2337/dc12-0337. PMID: 23069587

[57] Zhang, P., Gregg, E. W., Williamson, D. F., Barker, L. E., Thomas, W., Bullard, K. M., & Albright, A. (2010). A1C level and future risk of diabetes: A systematic review. *Diabetes Care*, *33*(12), 2753–2761. https://doi.org/10.2337/dc10-1154. PMID: 20974774; PMCID: PMC2977950

M20CCC.docx

原创性报告

22%	18%	19%	6%
相似指数	网际网络来源	出版物	学生文稿

主要来源

1	www.frontiersin.org<br>网际网络来源	2%
2	Submitted to The Hong Kong Polytechnic University<br>学生文稿	1%
3	bmjopen.bmj.com<br>网际网络来源	1%
4	bmcpsychiatry.biomedcentral.com<br>网际网络来源	1%
5	Basma Elsayed, Ahmed Alksas, Mohamed Shehata, Ali Mahmoud et al. "Exploring Neoadjuvant Chemotherapy, Predictive Models, Radiomic, and Pathological Markers in Breast Cancer: A Comprehensive Review", Cancers, 2023<br>出版物	1%
6	www.researchsquare.com<br>网际网络来源	1%
7	bmcpublichealth.biomedcentral.com<br>网际网络来源	1%
8	www.mdpi.com<br>网际网络来源	1%
9	Xinping Yu, Sheng Tian, Lanxiang Wu, Heqing Zheng, Mingxu Liu, Wei Wu. "Construction of a depression risk prediction model for type 2 diabetes mellitus patients based on NHANES 2007–2014", Journal of Affective Disorders, 2024<br>出版物	1%

10	pmc.ncbi.nlm.nih.gov<br>网际网络来源	1 %
11	Pushpa Choudhary, Sambit Satpathy, Arvind Dagur, Dhirendra Kumar Shukla. "Recent Trends in Intelligent Computing and Communication", CRC Press, 2025<br>出版物	<1 %
12	annalsmedres.org<br>网际网络来源	<1 %
13	peerj.com<br>网际网络来源	<1 %
14	Jingyu He, Sudan Liu, Menghan Jia, Manting Sha, Huijuan Rong, Zhengli Kang. "Effects of Preventive Nursing Care Combined with Auricular Acupressure on the Incidence of Postpartum Urinary Retention in Women with Vaginal Delivery", Journal of Modern Nursing Practice and Research, 2022<br>出版物	<1 %
15	www.science.gov<br>网际网络来源	<1 %
16	Bejoy Abraham, Madhu S. Nair. "Computer-aided detection of COVID-19 from CT scans using an ensemble of CNNs and KSVM classifier", Signal, Image and Video Processing, 2021<br>出版物	<1 %
17	Submitted to University of Bradford<br>学生文稿	<1 %
18	doaj.org<br>网际网络来源	<1 %
19	research.rug.nl<br>网际网络来源	<1 %

20	Yi Li, Bing Ran, Qihang Ye, Huisheng Zhong et al. "Lysine-polydopamine nanoparticles for ameliorating acidic microenvironment and neuroprotection in spinal cord injury repair", Biomaterials Advances, 2025<br>出版物	<1%
21	bmcanesthesiol.biomedcentral.com<br>网际网络来源	<1%
22	Submitted to The Cairnmillar Institute<br>学生文稿	<1%
23	Xiaohe Lin, Xiangyu Zhao, Yue Zeng, Lina Guo, Hongqvn Liu, Cui Mao, Ping Li. "Interpretable Machine Learning Models for Identifying Premenstrual Syndrome and Related Factors in Young Adult Women", Springer Science and Business Media LLC, 2025<br>出版物	<1%
24	pubmed.ncbi.nlm.nih.gov<br>网际网络来源	<1%
25	pdfcookie.com<br>网际网络来源	<1%
26	Chen Huang, Yanling Yue, Zimao Wang, Yong-Jin Liu, Nisha Yao, Wenting Mu. "Prediction of first attempt of suicide in early adolescence using machine learning", Journal of Affective Disorders, 2025<br>出版物	<1%
27	McDaniel, Claire. "Predicting Augmented Reality Virtual Try-On Adoption Using Machine Learning.", National University<br>出版物	<1%
28	Qi Huang, Denghong Wang, Shanshan Chen, Lei Tang, Chaoyang Ma. "Association of METS-IR index with depressive symptoms in US	<1%

adults: A cross-sectional study", Journal of Affective Disorders, 2024
出版物

29	Weiming Yan, Yan Sun, Yutong Wang, Yubin Liu, Weihua Yan, Dongling Li, Meizhu Chen. "The association of the serum levels of aldehydes with diabetes-related eye diseases: a cross-sectional population-based study", Environmental Science and Pollution Research, 2023<br>出版物	<1 %
30	www.nature.com<br>网际网络来源	<1 %
31	ijmedph.org<br>网际网络来源	<1 %
32	www.researchprotocols.org<br>网际网络来源	<1 %
33	Biwu Xu, Kaiyuan Li, Xiaowen Wang, Lei Wang, Yilong Man, Peng Liu. "The epidemiological burden of major depressive disorder in women of childbearing age and its relationship with the sociodemographic index: A systematic analysis based on the global burden of disease study from 1990 to 2021", Journal of Affective Disorders, 2025<br>出版物	<1 %
34	Cheng-Xi Li, Jing Leng, Kun Xiang. "Association of lifestyle behaviors and oral health care needs: Mediating effects of inflammatory markers", Preventive Medicine, 2024<br>出版物	<1 %
35	Jian Huang, Zhuoran Li, Xiaozhu Liu, Lirong Kuang, Shengxian Peng. "Development and validation of a web-based dynamic nomogram to predict individualized risk of severe carotid artery stenosis based on digital	<1 %

subtract angiography", Frontiers in Neurology, 2025
出版物

36	academic-accelerator.com<br>网际网络来源	<1 %
37	referencecitationanalysis.com<br>网际网络来源	<1 %
38	wagner.radford.edu<br>网际网络来源	<1 %
39	Arnaud Fondjo Kouam, Saturine Mengwe Mofor, Madeleine Yvanna Nyangono Essam, Armelle Gaelle Kwesseu Fepa et al. "Abnormal Serum Levels of Liver Enzyme Markers and Related Risk Factors in Type 2 Diabetes Mellitus Patients Attending the Buea Regional Hospital, Cameroon", Springer Science and Business Media LLC, 2025<br>出版物	<1 %
40	Jie Zhu, Haiyan Fu, Angela Y. M. Leung, Yining Zhang et al. "Exploring the barriers to the development of organizational health literacy in health institutions to meet the needs of older patients from multiple perspectives: a mixed-methods study", BMC Geriatrics, 2024<br>出版物	<1 %
41	Nan Wang, Xinyi Yan, Kellie Imm, Tianlin Xu, Shuang Li, Julia Gawronska, Ruixuan Wang, Lee Smith, Lin Yang, Chao Cao. "Racial and ethnic disparities in prevalence and correlates of depressive symptoms and suicidal ideation among adults in the United States, 2017–2020 pre-pandemic", Journal of Affective Disorders, 2024<br>出版物	<1 %
42	Perihan Ozkan Gumuskaya, Ozgur Altun, Emine Yildirim, Nur Karakutuk Yuztas et al.	<1 %

"The Association Between Depression and Antidiabetic Treatments in Type 2 Diabetes Patients with Both Good and Poor Glycemic Control", Journal of Clinical Medicine, 2025
出版物

43	Wesley Lo, Senbao Lu, Dmitry Korkin, Angela C. Incollingo Rodriguez, Lourah M. Kelly, Jean A. King, Benjamin C. Nephew. "Machine learning based prediction of high school student mental health", Cold Spring Harbor Laboratory, 2025<br>出版物	<1 %
44	Yan Huang, Lin Han, Yefei Xiao, Ruiqi Wang, Dan Liu, Bing Cao. "Mediating role of depression in the relationship between allostatic load and mortality", Psychoneuroendocrinology, 2025<br>出版物	<1 %
45	Zhengyuan Yan, Lili Chang, Shuang Sun, Zhongwen Sun. "Geniposide attenuates obesity-related depression: involvement of decreased neuroinflammation and synaptic engulfment", NeuroReport, 2025<br>出版物	<1 %
46	discovery.ucl.ac.uk<br>网际网络来源	<1 %
47	neptjournal.com<br>网际网络来源	<1 %
48	www.imrpress.com<br>网际网络来源	<1 %
49	www.ncbi.nlm.nih.gov<br>网际网络来源	<1 %
50	Han Wang, Suyan Tian, Sen Bai, Chunqi Yang, Zhengang Jiang, Nannan Li. "Associations Between Thyroid Function and Periodontitis:	<1 %

A Machine Learning Approach Using NHANES", International Dental Journal, 2025
出版物

51	Submitted to King's College<br>学生文稿	<1 %
52	boa.unimib.it<br>网际网络来源	<1 %
53	discovery.researcher.life<br>网际网络来源	<1 %
54	dspace.library.uu.nl<br>网际网络来源	<1 %
55	lipidworld.biomedcentral.com<br>网际网络来源	<1 %
56	www.dovepress.com<br>网际网络来源	<1 %
57	www.nutricionhospitalaria.org<br>网际网络来源	<1 %
58	Changfeng Guo, Haoran Zhou, Ivan Miguel Pires, Paulo Jorge Coelho, Runzhe Tong, Farnaz Farid. "Towards an explainable machine learning model to reduce readmission risks for diabetes patients", Informatics in Medicine Unlocked, 2025<br>出版物	<1 %
59	Farraj, Sinan Abi. "Improving Removal and Monitoring of Nanoplastics and Microplastics in Aggregation-Based Wastewater Treatment Systems", McGill University (Canada), 2024<br>出版物	<1 %
60	Ron Stout, Daniel Reichert, Rebecca Kelly. "Lifestyle Medicine and the Primary Care Provider - A Practical Guide to Enabling Whole Person Care", CRC Press, 2025<br>出版物	<1 %

61 Qiqi Yan, Guiling Liu, Ruifeng Wang, Dandan Li, Deguang Wang. "Development and validation of a nomogram for predicting depression risk in patients with chronic kidney disease based on NHANES 2005–2018", Journal of Health, Population and Nutrition, 2025 <1%

出版物

不含引文	关闭	不含相符结果	关闭
排除参考书目	开		
